# A tempo-spatial controllable microfluidic shear-stress generator for in-vitro mimicking of the thrombus

**DOI:** 10.1186/s12951-024-02334-6

**Published:** 2024-04-17

**Authors:** Zhihang Yu, Yiqun Chen, Jingjing Li, Chang Chen, Huaxiu Lu, Siyuan Chen, Tingting Zhang, Tianruo Guo, Yonggang Zhu, Jing Jin, Sheng Yan, Huaying Chen

**Affiliations:** 1 School of Mechanical Engineering and Automation, Harbin Institute of Technology, Shenzhen, Shenzhen, 518055 China; 2 Graduate School of Biomedical Engineering, The University of New South Wales, Sydney, NSW 2052 Australia; 3 Institute for Advanced Study, Shenzhen University, Shenzhen, 518060 China

**Keywords:** Biotechnology Microfluidics, Shear-stress generator, Cell stress, Thrombus model

## Abstract

Pathological conditions linked to shear stress have been identified in hematological diseases, cardiovascular diseases, and cancer. These conditions often exhibit significantly elevated shear stress levels, surpassing 1000 dyn/cm^2^ in severely stenotic arteries. Heightened shear stress can induce mechanical harm to endothelial cells, potentially leading to bleeding and fatal consequences. However, current technology still grapples with limitations, including inadequate flexibility in simulating bodily shear stress environments, limited range of shear stress generation, and spatial and temporal adaptability. Consequently, a comprehensive understanding of the mechanisms underlying the impact of shear stress on physiological and pathological conditions, like thrombosis, remains inadequate. To address these limitations, this study presents a microfluidic-based shear stress generation chip as a proposed solution. The chip achieves a substantial 929-fold variation in shear stress solely by adjusting the degree of constriction in branch channels after PDMS fabrication. Experiments demonstrated that a rapid increase in shear stress up to 1000 dyn/cm^2^ significantly detached 88.2% cells from the substrate. Long-term exposure (24 h) to shear stress levels below 8.3 dyn/cm^2^ did not significantly impact cell growth. Furthermore, cells exposed to shear stress levels equal to or greater than 8.3 dyn/cm^2^ exhibited significant alterations in aspect ratio and orientation, following a normal distribution. This microfluidic chip provides a reliable tool for investigating cellular responses to the wide-ranging shear stress existing in both physiological and pathological flow conditions.

**Graphical Abstract:**

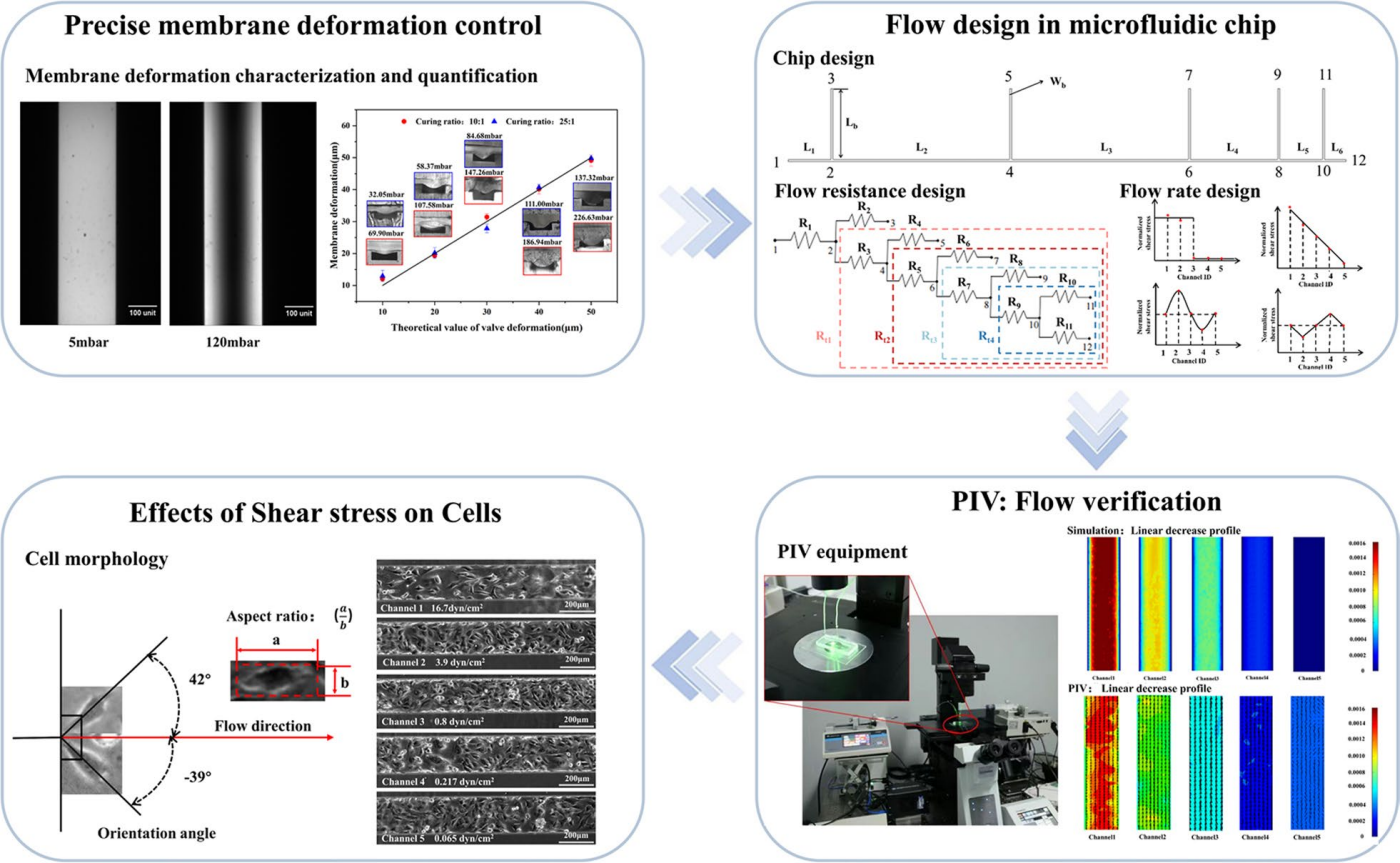

**Supplementary Information:**

The online version contains supplementary material available at 10.1186/s12951-024-02334-6.

## Introduction

The entire vascular system is exposed to mechanical forces such as stretch, strain and shear stress due to the blood flow [1]. The wall shear stress exerted on the endothelial wall of the vessel is the mechanical frictional force parallel to the blood flow. The magnitude of physiological shear stress may vary from 1 to 70 dyne/cm^2^ depending on the type of vessel [2] and developmental ages [3]. Typically, it is accepted that the shear stress within human veins falls within the range of 1–6 dyn/cm^2^, while in arteries, it is within the range of 10–70 dyn/cm^2^ [4]. It regulates cell morphology [5], proliferation [6], differentiation [7], migration [8] and other cell behaviors [1] involved in developmental and physiological vascular processes [9–11]. Dysregulated pathological shear stress has been found in hemopathy [12], cardiovascular disease [13] and cancer [14]. For example, the shear stress may be greater than 1000 dyne/cm^2^ in a 95% constricted artery stenosis [2]. Simultaneously, elevated shear stress can induce mechanical damage to endothelial cells. Endothelial cells subjected to prolonged exposure to low shear force levels, specifically below 3 dyn/cm^2^, are significantly associated with an elevated risk of atherosclerosis [15]. Therefore, understanding the role of shear stress on vascular cells can provide cues for pathogenic diagnosis, and treatment of cardiovascular related diseases [16, 17].

Microfluidics has become the most promising technique for generating both physiological and pathological shear stress owning to its advantages in accurate fluid control [18–20], biocompatibility and microenvironment mimicking [21–23]. Microfluidic devices have unveiled the influence of physiological shear stress on various cell morphological parameters, including cell alignment [24], cytoskeleton [25] and intracellular actin arrangement [26]. Additionally, these devices have been crucial in studying endothelial cell and leukocyte adhesion [27], migration [8, 28], viability and proliferation [6]. Furthermore, the microfluidic-generated shear stress has demonstrated ability to induce stem cell differentiation [6] into endothelial cells [29], muscle cells [30], and bone cells [7, 31]. Moreover, the pathological shear stress has shown an important role in disease progression by up-regulating gene expression of inflammatory cytokines [13, 32], thrombomodulin mRNA [33], reactive oxygen species (ROS) [34] et al. Microfluidic methods generating shear stress can be categorized into either active or passive ones. The passive ones produce the flow and shear stress gradient by changing the geometry (the length [35, 36], width [37, 38] and shape [39]) of the channel. For example, the microfluidic chips with triangular and trapezoidal cross-sectional shapes enable the generation of shear stress gradients within the same section [40, 41]. Additionally, a microfluidic chip using circuit analogy is proposed to create a wide range of shear stress gradients (1–1000 times) [7, 42]. However, the passive method results in an inherent limitation: once the chip is fabricated, the gradient cannot be adjusted, rendering it inflexible. In contrast, the active techniques can dynamically control not only the magnitude but also the type, such as the unidirectional, pulsating, oscillating, and turbulent flow [43]. Active shear stress generation methods usually employ the micropumps and microvalves based on their driving mechanisms [1]. The most commonly used ones rely on the microvalves integrated on microfluidic chips to control shear stress. For instance, through precise control of the microvalve, the chip can generate shear stress ranging from 0.4 to 15 dyn/cm^2^ [29] to investigate the crucial shear influence on the development of endothelial cells. Active methods can generate stable and reciprocating shear stress, which may better mimics the dynamic physiological fluid flow (eg, cardiac pulsatile flow [44–46]) in the cardiovascular system.

The human pathological shear stress may be as high as 1000 dyn/cm^2^. Therefore, to simulate the full physiological and pathological shear stress environment, the device needs to have the ability to generate continuous and high gradient shear stress. Compared with the active method (0.4–15 dyn/cm^2^) [29, 41] in the existing microfluidic technology, the passive method can generate a larger shear stress gradient (0.01–10 dyn/cm^2^ and 1–1000 times variation) [7]. However, the passive method cannot flexibly adjust the shear stress after the chip is fabricated. This means photolithography moulds should be fabricated for different shear stress combination. Additionally, the shear stress generated by the passive method is discrete in different regions. Existing microfluidic technology faces significant limitations in generating a wide range of shear stress, adjusting flexibility, and adapting spatially and temporally. Consequently, accurately and comprehensively simulating and visualizing shear stress microenvironments within the human body remains challenging. This obstacle hinders a thorough understanding of physiological phenomena and pathologies like thrombosis. To address this, an urgent need arises for a technology capable of faithfully replicating all shear stress environments in the human body, enabling research on the physiological and pathological impacts of shear stress.

**Fig. 1:**
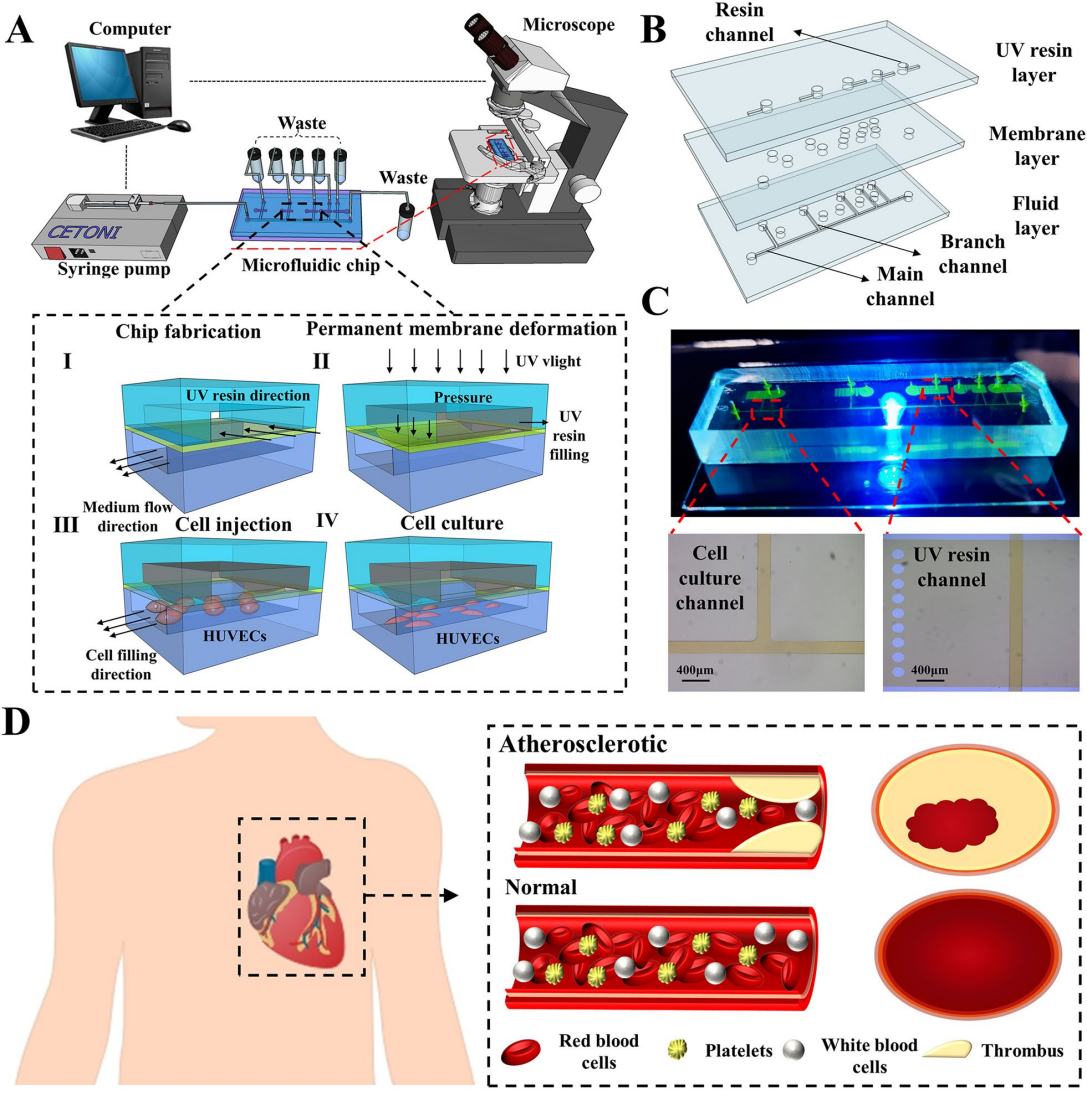
The microfluidic chip to generate various combinations of shear stress. **A** The diagram of the microfluidic chip and the imaging system. The inset shows the fabrication and operation diagrams. **B** The exploded view of the chip with three layers; **C** the photo of the microfluidic chip filled with fluorescein to highlight channels; **D** the thrombosis model in humans

In this paper, we present a microfluidic chip capable of generating a comprehensive range of shear stress, encompassing both physiological and pathological conditions found within the human body. The chip accomplishes precise control of the membrane by coupling the pressure field and the flow field, enabling flexible manipulation of shear stress generation. Additionally, the chip allows for the simulation of pathological thrombus flow (as shown in Fig. 1D). The membrane on each branch channel can be permanently deformed by UV cured resin to create precise channel constriction. By adjusting the constriction in five channels, the flow resistance in the chip varied to generate the shear stress combination up to 929 times. The microfluidics chip fluid dynamics design enables the simultaneous generation of five different magnitudes of shear stress within its five channels, effectively spanning the complete spectrum of physiological and pathological shear stress observed in the human body. By integrating control over the channel inlet flow rate, precise spatial and temporal control of the shear stress within the chip can be achieved. Shear influence on the human umbilical vein endothelial cells (HUVECs) were characterized based on morphological (orientation angle and aspect ratio) and adhesiveness change. This device may be of extensive application potentials in the fields of modelling the influence of large shear stress on cells, e.g., regulation of cellular development, disease pathogenesis.

## Materials and methods

### Device design and manufacture

A microfluidic chip (Fig. 1A) was developed to generate various combinations of shear stress in five branch channels with distinct and variable local cross section. The chip consisted of three layers (Fig. 1B and C): a top layer containing five individual channels (7 mm long, 3 mm wide and 50 μm high) for UV resin (BV007, miicraft, China) filling, a middle layer with a 10-μm thick membrane, and a bottom layer including a main channel (200 μm wide and 50 μm high) splitting to five branch channels (8 mm long, 200 μm wide, and 50 μm high) spaced at different distance along the main channel (Additional file 1: Figure S1A). The channels on the top layer were filled with resin cured at a given injection pressure to create permanent deflection of the membrane towards the branch channels on the bottom layer. By varying the injection pressure of resin, it is flexible and straightforward to create different local constriction (50 μm high) in the branch channels. The constriction was able to adjust the flow distribution and the shear stress in five branch channels which were employed for long-term culture of endothelial cells.

The molds (50 μm high) of the top and bottom layer were manufactured by photo lithography using SU8-2025 (SU8-2025, Microchem, USA) as reported elsewhere [47–49]. Then the PDMS replicas of both layers were molded using Sylgard 184 elastomer base and curing agent (Sylgard 184, Dow corning, USA) with the weight curing ratio (elastomer base: curing agent) of 10:1 [50–52]. The uncured PDMS with curing ratio of either 25:1 or 10:1 was spin-coated at 5500 rpm on blank wafer to form a membrane with a thickness of approximately 10μm before complete baking. After punching of the inlet and outlet ports on both bottom and top layers, the top layer was bonded to the membrane following plasma etching for 30 s. Afterward, the top layer with membrane was peeled off and bonded to the bottom layer after plasma etching. Then the UV curable resin was filled into the five individual channels (on top layers) using a precision pressure controller at pressure from 0 to 500 mbar (EasyMicrofluidics, Dalue Lab, China). Afterward, the resin was exposed to UV light (UVS30-120, Runzhu, China) for 15 min to create permanent membrane deformation (0–50 μm) before the pressure was released. The tubing with the inner/outer diameter of 0.51/1.52 mm was securely attached to the inlet and outlet ports using syringe needles with a flat-end of 21-gauge. Finally, the devices were stored for the following experiments.

### Membrane deformation and resulted flow resistance

The membrane deformation was firstly evaluated by imaging the intensity of the fluorescence which was altered by the height of the constricted channels. The fluorescein (F104053, Aladdin, China) was dissolved in an alkaline ionic buffer (Tris Buffer, Macklin, China) to the mass concentration of 3%, before being injected into the microfluidic chip at 20 μL/min using a syringe pump (CETONI, Germany). Following the complete filling of the flow channels, the fluorescein was kept static. The channel with fluorescein under the deformed membrane (with the curing ratio of either 10:1 or 25:1) was imaged using a microscope (IX 73, Olympus, Japan) equipped with a 450 nm laser and a CCD camera (C11440-36U, Hamamatsu, Japan). The temporary deformation of the membrane was generated using the pressure from 10 to 500 mbar with the increasement of 10 mbar. To validate the experimental accuracy, Comsol was employed for simulating the membrane deformation under various pressure conditions. Refer to Additional file 1: Figure S2 and Table S1) for detailed information.

The membrane deformation was further examined by direct imaging of the cross section created by the cured UV resin. According to fluorescence imaging, to acquire the deformation of 10–50 μm, the pressures applied to the membrane of the curing ratio of 25:1 and 10:1 were 32.1–137.3 mbar and 69.9–226.6 mbar, respectively (Additional file 1: Table S2). After UV curing, the branch channels were cut through the membrane region. The cross section was imaged using a microscope with the objective of X10. The deflection of the membrane was measured using ImageJ [48, 53].

Additionally, the 3D models of part of the branch channel were created in COMSOL to evaluate the flow resistance when the membrane deflected for 0, 20, 30, 40 and 50 μm (Additional file 1: Figure S3A and B). Refer to the Additional file 1 for the relevant model meshes and corresponding results (Additional file 1: Table S3). The laminar flow module at steady state with the inlet speed of 0.00278m/s was employed in simulation. The outlet was set to 0 Pa and all other boundaries were non slip walls. The pressure drop ($\Delta P$) along the constriction region and flow rate ($Q$) were obtained from the simulation to calculate the hydraulic resistance (${R}_{h}$) of both the whole branch channel and the constriction region using the equation of ${R}_{h}=\Delta P/Q$.

### Predication of shear stress combinations

The device dimension and the flow resistance of the constriction region was analyzed to create various combinations of shear stress in five branch channels. As shown in Additional file 1: Figure S1B, the microchannels of the whole device were equivalent to a electrical circuit. The flow rates in the five branch channels were closely related to the length of the branch channels and their spacing along the main channel. For a given device design, if the hydraulic resistance of the constriction region varied, the flow rate through each branch channel might change significantly. The flow resistance of the channel with rectangular cross section is calculated by [1, 54]:

1 $$R_{h} = \frac{12L\mu }{{wh^{3} \left( {1 - 0.63*h/w} \right)}}$$

where L, w and h are the length, width and height of the channel, and $\mu$ is the dynamic viscosity of the liquid. The flow resistance of the whole branch channel was 4.46 × 10^12^, 5.27 × 10^12^, 6.42 × 10^12^, 8.84 × 10^12^, 12.24 × 10^12^, 16.00 × 10^12^ N·s·m^−5^ when the membrane deformed for 0, 10, 20, 30, 40, and 50 μm, respectively.

The shear stress ($\tau$) in the microchannel is given by:

2 $$\tau = 8\varphi \left( n \right)\frac{\mu Q}{{D_{h} A}}$$

where Q is the flow rate, *D*_*h*_ ($2wh/(w+h)$) is the hydraulic radius, n ($h/w$) is the aspect ratio of the channel, and *φ(n)* is the correction factor given by:

3 $$\varphi n = \left[ {\frac{{n^{2} + 1}}{{\left( {n - 1} \right)^{2} }} - \frac{{\left( {n + 1} \right)}}{{\left( {n - 1} \right)lnn}}} \right]^{ - 1}$$

Thus the Additional file 1: Equations S1–S14, of the shear stress in branch channels shown in Additional file 1 were solved using Matlab (Matlab2016a, MathWorks, USA) to obtain the related shear stress combinations as the membrane deflection varied from 0 to 50 μm and the inlet flow rate was 1.67 μL/min.

### Numerical and experimental studies of the shear stress

Both 2D and 3D models of the whole device were created in COMSOL to study the velocity and shear stress in branch channels either without or with membrane deformation, respectively. In the former model, the inlet flow was set to 0.00278 m/s, and all outlets of both the branch and the main channels were set to 0 Pa. In the latter model, the membrane deformation from the first to the fifth branch channels was set to 50, 40, 10, 0, 50 μm, respectively. The inlet flow was also 0.00278 m/s and all outlets were set to 0 Pa. The details of the simulation were reported in Additional file 1.

Besides simulation, the flow through the branch channels was examined using micro particle imaging velocimetry (µPIV). The devices either without or with the channel constriction identical to that in the simulation section were mounted on the stage of a microscope (Olympus IX73, Olympus, Japan) with high-speed camera (Phantom V7.3, Phantom, USA). The fluorescent particles (excitation wavelength: 450nm; emission wavelength: 520nm) with a diameter of 2 μm were diluted 100 times with deionized water. Then they were injected into the chips at 1.67 μL/min (0.00278m/s) using a syringe pump (CETONI BASE 120, CETONI, Germany). After the flow was steady, the flowing particles in five branch channels were excited using a laser (MBL-FN-473nm, CNI, China) while imaged with the frame rate of 1000 per second. The flow field was calculated from the pair of images using Fast Fourier Transform (FFT) windows deformation in the software of PIVlab (Matlab2016a, MathWorks, USA). The measurement was performed 100 times for each position in to obtain the average velocity.

### Shear-induced detachment of endothelial cells

The microfluidic chip with one channel (200 μm wide and 50 high) bonded on a glass slide was fabricated to examine the adhesion force of endothelial cells. The microchannel was firstly sterilized 75% ethanol (Ante, Anhui, China) injected at 1 μL/min for 30 min. Then the channel was rinsed using DI water at 1 μL/min for 10 min. After the chip was placed in an incubator (Heracell™ VIOS 250i, Thermo, USA) at 37 ± 0.1 °C with 5% CO_2_, the channel was injected with 1 mg/mL fibrin (F914989, MACKLIN, China) at 1 μL/min for 30 min to enhance adhesion of cells. Then the suspension of HUVECs (10^6^ cells/mL) was manually injected into the chip, before DMEM with 10% FBS and 1% PS (referred to media without stated otherwise) was perfused at 0.1 μL/min for 0–72 h. When the microchannel was confluence, the device was moved out of the incubator and mounted on the microscope (with a X10 objective) with a stage-top incubator at 37 ± 0.1 °C with 5% CO_2_ and a CCD camera. Meanwhile, the media flow rate was set to 100 μL/min and increased 100 μL/min every 4 min to the flow rate of 600 μL/min. This allowed the cells in the channel to be exposed to shear stress of 166.7, 333.4, 500, 666.7, 833.5, and 1000 dyn/cm^2^ for four minutes under each shear. Meanwhile, the cells on the channel floor were imaged every 2 min to record the cell detachment process.

Then 24-h exposure of cells to various shear stress was performed. After culturing HUVCEs to confluence as mentioned above, the chip was placed in a stage top incubator (Tokai Hit, Shizuoka-ken, Japan) at 37 ± 0.1 °C with 5% CO_2_. Then the inlet flow was adjusted to either 10 (16.7 dyn/cm^2^), 5(8.3 dyn/cm^2^), or 0.1 (0.17 dyn/cm^2^) μL/min for a 24-h duration. The cells were imaged every 2 min for 24 h to record the growth under shear stress.

### Shear influence on endothelial cells

The devices both without constriction and with the membrane deformation of 50, 40, 10, 0, 50 μm (Channel 1–5) were employed to study the influence of large-range shear stress non HUVECs. After the chip was mounted on the microscope ( CytoScanner, Dalue Lab, China), the microchannel was sterilized, rinsed using DI water and primed with fibrin using protocols mentioned in the former section. Then suspension of HUVECs (10^6^ cells/mL) was manually loaded into the chip using a syringe to ensure at least one third of the microchannel surface was occupied when the they attached and fully spread. Then the chip was placed inside an incubator at 37 ± 0.1 °C with 5% CO_2_. The media was injected into inlet at 0.1 μL/min for 5 days to let the cells grow to confluency in the branch channels. Then the flow rate was raised to 13.07 μL/min and 25.27 μL/min for the devices without and with constriction, respectively. The flow rate was maintained for 24 h to expose the cells to designed shear stress that was significantly larger than that during the culture in the incubator. The shear stress in Channel 1 to 5 in the device without constriction was 16.7, 3.9, 0.8, 0.217, and 0.064 dyn/cm^2^, respectively. While that was 16.7.10.3, 8.0, 2.6 and 0.153 dyn/cm^2^ for Channel 1 to 5 in the device with constriction.

### Data and image processing

Briefly, the Imagej [55] was employed to assess the fluorescent images and the cell number in the studies of both adhesion and morphology (size and orientation angle). The SPSS statistics (IBM, Newyork, USA) was employed to perform normal distribution test on the cell positioning angles based on Kolmogorov Smirnov (K–S). One-way ANOVA with the p value of 0.05 was performed to analyze the difference of the data. Error bars represent the standard deviation of at least three replicates.

## Results and discussion

### Membrane deformation and resulted flow resistance

**Fig. 2:**
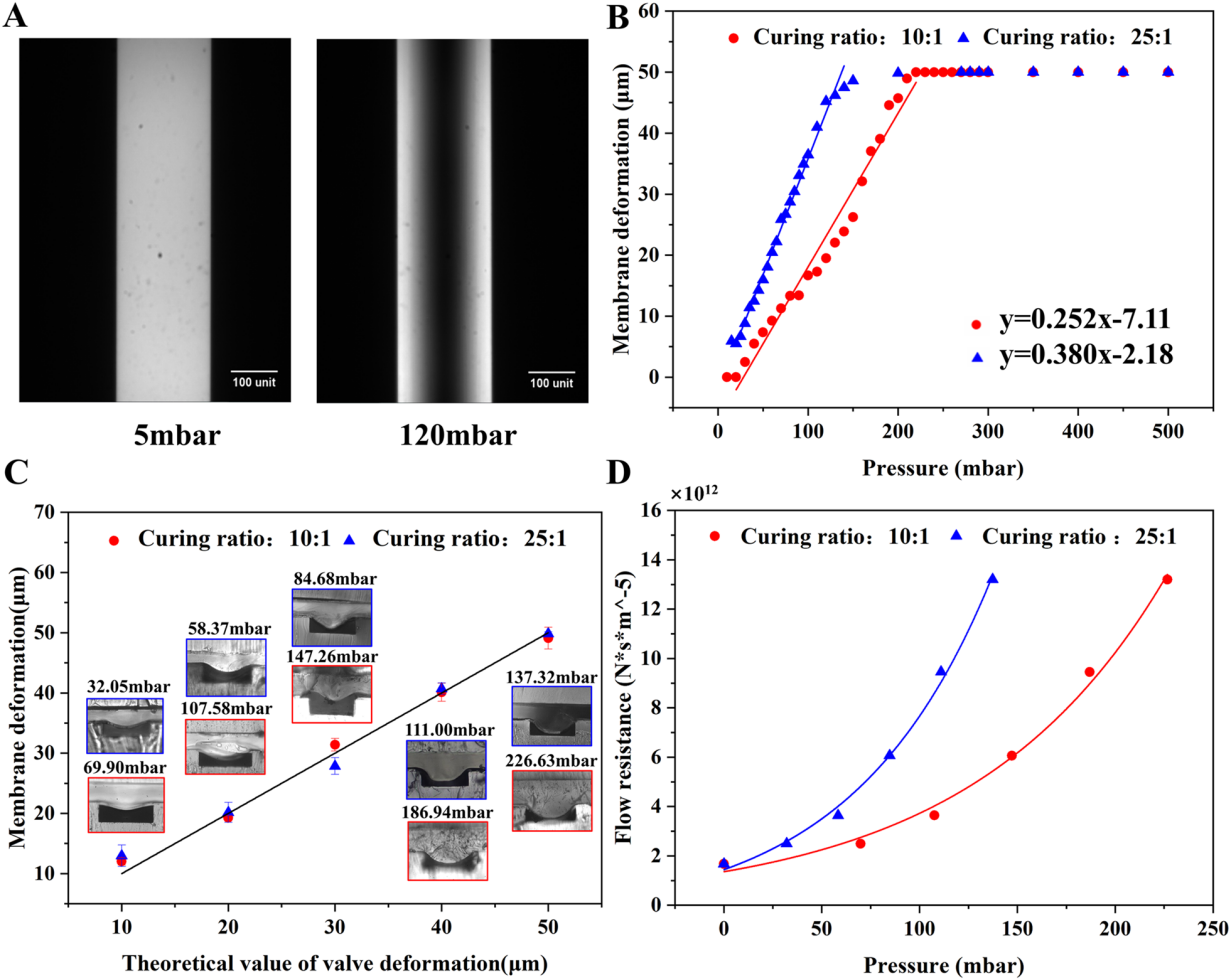
The studies of membrane deformation and flow resistance. **A** The microscopic images of the fluorescent liquid in channels with the membrane (curing ratio of 25:1) under distinct pressure. The deformation of membrane under various pressure determined by **B** fluorescence imaging and **C** cross section imaging. **D** The flow resistance of the constricted region when the membrane deflected 10–50 μm at various pressure

The relative degree of membrane deformation was indirectly quantified by characterizing the fluorescence intensity. As the deflection of the membrane, the volume and the intensity of the fluorescence liquid under the membrane decreased. Figure 2A shows the fluorescence images of channels with the membrane (curing ratio of 10:1) deflected under 5 mbar and 120 mbar. It was evident that irrespective of the curing ratio, a linear relationship existed between the membrane deformation (y) and the applied pressure (x) until the membrane deflected 50 μm to contact the channel floor (Fig. 2B). The linear regression models were y = 0.258x − 7.11 and y = 0.380x − 2.18 for the curing ratio of 10:1 and 25:1, respectively. The linear correlation reveals that the membrane adheres to Hooke's law, which states that stress and strain in a material exhibit a linear relationship within the elastic range. Additionally, the fitted line associated with the 25:1 membrane had a larger slope, since it possessed a smaller Young’s modulus. This 25:1 membrane was more susceptible to deform owing to its smaller Young's modulus.

To validate the experiment's accuracy, comsol software was employed to simulate the membrane deformation under varied pressure conditions. Detailed results are presented in Additional file 1: Figure S2. Additional file 1: Figures S2E and H reveal a remarkable concordance between the simulated scatter plots of membrane deformation under different pressure conditions and the experimental fitting curves. Through a comprehensive synthesis of simulation and experimental data, the corresponding pressure was obtained for membrane deformations of 10, 20, 30, 40, and 50 μm (see Additional file 1: Table S2).

Figure 2C illustrates the cross-sectional photos of the constriction region and magnitude of deformation induced by the cured UV resin which were injected and cured at the pressure acquired from Fig. 2B. The deflection exhibited a parabolic shape with greater deformation in the middle and small deformation on both sides (Fig. 2C). Additionally, the membrane deformation was of small errors under the same pressure condition. It revealed that the actual deformation caused by resin closely resembled the pneumatic driven deformation measured using the fluorescent liquid. Therefore, it is possible to predict the exact pressure inducing the desired membrane deformation using the regression models in the Fig. 2B.

Given the irregular constriction of the channel owing to the membrane deformation, it was crucial to simulate the flow within the deformed channel to obtain flow resistance. As depicted in Fig. 2D and Additional file 1: Figure S3D, the flow resistance of the constriction region was proportional to the pressure (or the membrane deflection) regardless of the curing ratio of the membrane. For the given pressure, the membrane with less curing agent (25:1) resulted in larger flow resistance, since it deflected to a larger extent in comparison to that with more curing agent (10:1).

The precise control of the flow resistance relies on the accurate deformation of the membrane. However, measurement of the deformation is challenging and mainly by either numerical simulation or imaging. For instance, simulation revealed that 2000 mbar was necessary to deflect a 20 μm thick film (with a curing ratio of 10:1) for by 30 μm [56, 57]. The confocal microscopy has been employed for a more intuitive visualization of membrane deformation [48]. It reported that under a pressure of 2000 mbar, a film with a thickness of 10 μm deforms for approximately 15 μm.

This paper measured the deformation by imaging both fluorescence liquid and the cross section. Although the former one was indirect visualization, both methods are accurate and repeatable. Additionally, the observed variations between the pressure for the membrane deflection in different articles can be attributed to several factors, for example, the differences in the membrane production process (such as curing ratio, curing temperature, and time), the thickness, width and length of the membrane.

### Predication of shear stress combinations

**Fig. 3:**
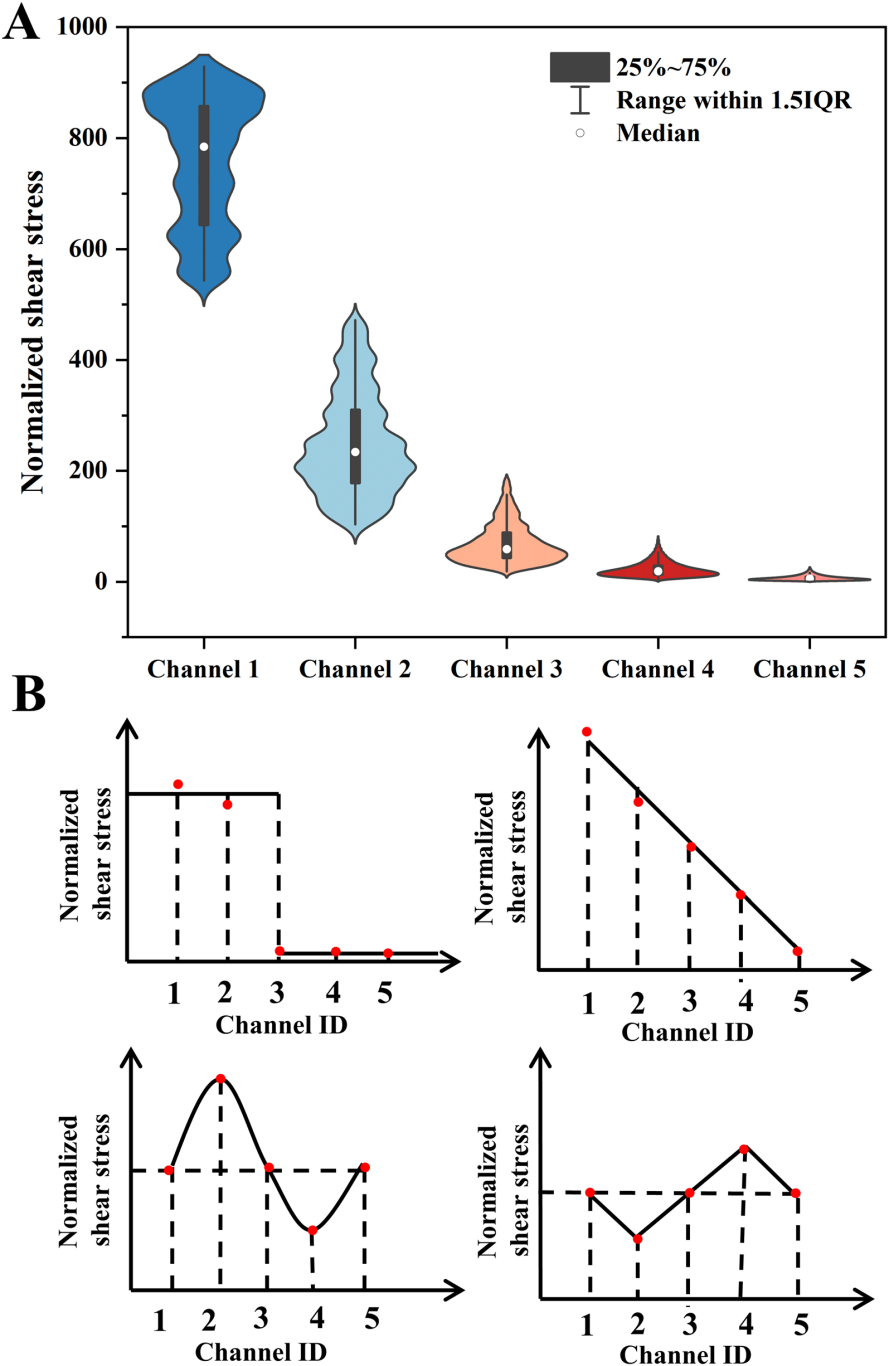
The theoretical study of the shear stress combinations in 5 channels. **A** The shear stress range in each channel when the membrane deformed independently for various extent. **B** The square, linear, sine and jaggies profiles of the shear stress in 5 channels

The flow resistance of microfluidic channels can usually be analogous to resistors connected either in parallel or in serials in digital circuit (see Additional file 1: Figure S1B). Additional file 1: Table S4 presents the calculated flow resistance values of different regions in the microfluidic chip. For the given inlet flow rate, the device was able to create 7776 flow rate combinations by varying the membrane deformation with the step of 10 µm in each branch channel. The shear stress (Fig. 3A) of the above combinations was calculated by Matlab and normalized to the minimal shear stress in Channel 5 when the membrane deflected for 0, 0, 0, 0 and 50 µm for the channel 1 to 5, respectively. The ratio between the maximum and the minimum shear stress generated by the device was 929 (Fig. 3A and Additional file 1: Table S5). In another word, the device was able to create shear stress with 1–929 times variation merely by varying the membrane deflection of 5 branch channels. For the given combination of deformations, the microfluidic chip generates maximum and minimum shear stress gradients of 874 and 20 times, respectively (see Additional file 1: Table S6).

Additionally, the device was able to create precisely designed shear stress profiles across branch channels by varying the degree of membrane deformation. For example, the square, linear, sine and Jaggise varied profile of shear stress in five branch channels (Fig. 3B). The required membrane deformation and the flow rates in the five branch channels were summarized in Additional file 1: Table S7. If the inlet flow rate was altered, the magnitude of the shear stress may increase in each branch channel, but the ratio of the shear stress was constant once the membrane deflection was fixed. For instance, if the inlet flow rate raised 100%, the magnitude of the shear stress in each channel doubled regardless of the profile of the combination (Fig. 3B). To modify the shear stress profile the membrane deformation combination should be altered.

Shear stress variation based on the alteration of local size along the channel was a straightforward and most frequently used method, it lacked the ability to dynamically adjust the once the SU-8 mould was manufactured. Hence, the microvalve is employed to dynamically control the cross-sectional shape, precisely regulating the flow resistance within the channel and enabling real-time adjustments to the shear stress within the chip [29]. Moreover, by dynamically controlling the valve deformation, the flow pattern can be precisely regulated to effectively simulate various flow microenvironments [44]. However, this method demands continuous pressure to maintain deformation of the microvalves, thereby increasing the complexity of the experimental process. The method in this paper enabled permanent membrane deformation using UV resin injected at a given pressure after the fabrication of the PDMS device. Although it was limited in the real-time adjustment of membrane deformation in the same PDMS device, it only required one SU-8 mould (by photolithography) and was able to manufacture devices with hundreds of deformation combinations. Therefore, it may be used to generate shear stress in a large range.

### Numerical and experimental studies of the flow field

Besides the calculation of shear stress based on analysis of the flow rates in the above section, it was further validated using both numerical simulation and µPIV measurement. The intersection areas between branch channels and the main channel from both simulation and experiments (Additional file 1: Figures S4 and S5) demonstrated significant effect of flow shunting regardless of the presence of the constriction. Due to the mass conservation nature, the flow through any section in the same branch channel was constant. The shear stress at the up and downstream of the constriction was identical. However, the constriction altered the flow resistance and thus the flow rate in all branch channels (see velocity contours in Additional file 1: Figure S6).

**Fig. 4:**
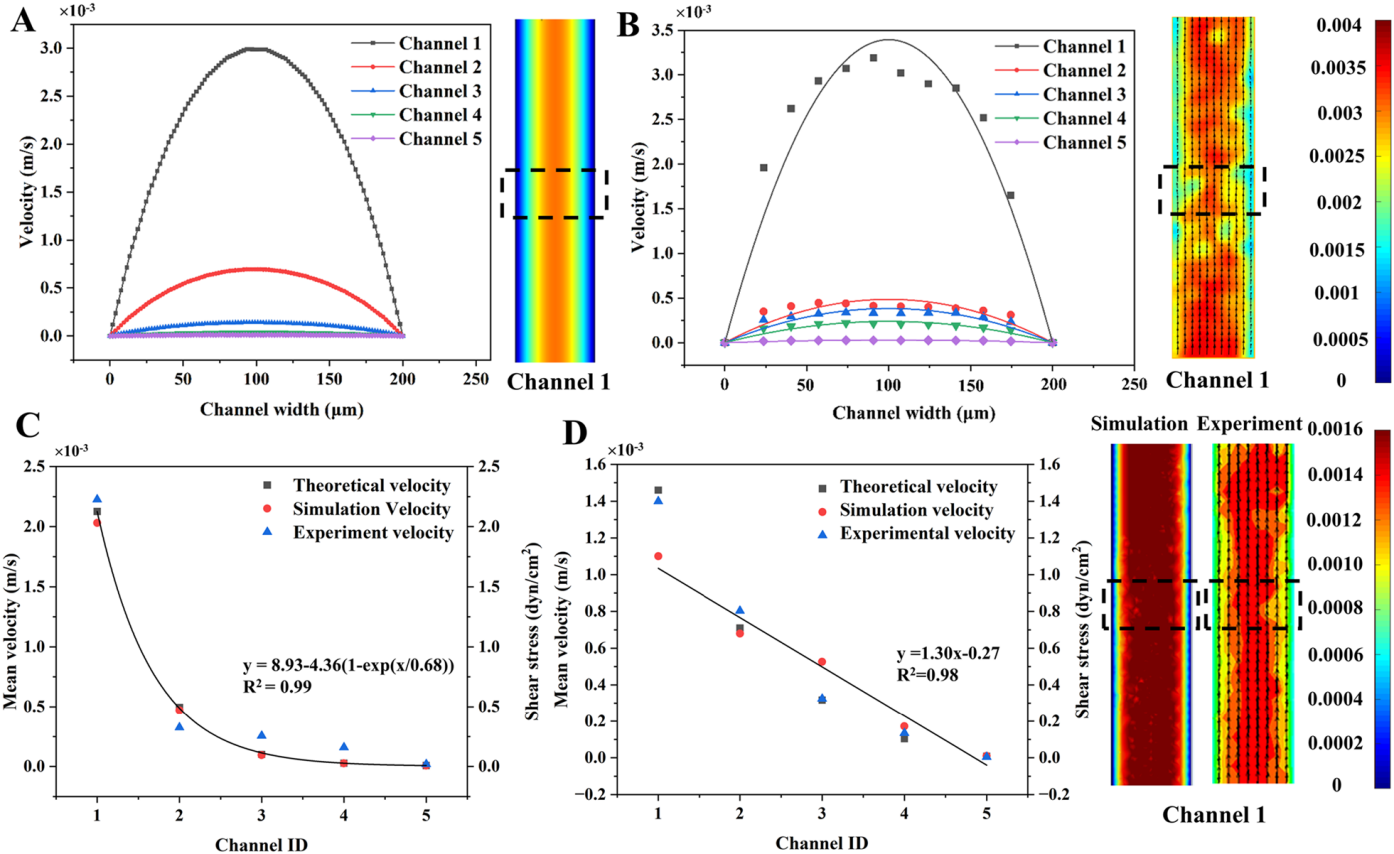
The study of the flow field in branch channels. The velocity profiles (across the channel width) acquired from **A** simulation and **B** µPIV measurement in the 5 channels without membrane deformation. **C** The velocity and shear stress in 5 branch channels without membrane deformation. **D** The flow velocity and shear stress in branch channels when the membrane deformed 50 μm, 40 μm, 10 μm, 0 μm and 50 μm in Channel 1 to 5, respectively. Insets showed the velocity contours from simulation and experiment

Figure 4A and B showed the velocity profiles obtained respectively by simulation and µPIV using the devices without constriction when the inlet flow rate was 1.67 µL/min (0.00278 m/s). The insets showed velocity contours and the regions (dotted boxes) for velocity calculation across the width of the channel. The velocity across the width was of a parabolic profile with maximum in the channel center due to the laminar flow nature. The lines in Fig. 4B represent the parabolic models (see Additional file 1: Table S8) fitted to the µPIV data. The R^2^ of all models ranged from 0.79 to 0.94, suggesting the velocity distribution across the channel met the characteristic of laminar flow.

The mean velocity of 5 channels in devices either without (Fig. 4C) or with constriction (Fig. 4D) were acquired by analytical calculation, simulation, and experiment. As the shunt effect of the branch channels in Fig. 4C, the mean velocity (shear stress) drastically decreased from 2.03 × 10^–3^ m/s (2.03 dyn/cm^2^) in Channel 1 to 7.09 × 10^–6^ m/s (7.09 × 10^–3^ dyn/cm^2^) in Channel 5 although the membrane was not deflected. The shear stress was determined by the Channel ID using an exponential decay model of y = 8.93 − 4.36(1 − exp(x/0.68)) (R^2^ = 0.99). However, the mean velocity (shear stress) linearly decreased 99.7% (99.7%) from Channel 1 to Channel 5 in devices with constriction (Fig. 4D). The relationship between shear stress and the channel ID was described by y = 1.30x − 0.27 (R^2^ = 0.98). The devices without and with constrictions were able to correspondingly generate 284.8- and 109.2-times shear stress variation across five channels. Thus, they were employed to examine the shear influence on HUVECs in the last section.

In both Fig. 4 C and D, there was 8.7–21.4% difference between the simulation and the experiment. However, the difference between the experiment and the analytical analysis was 11.6–14.4%. The variation between the velocity acquired by distinct methods might be related to the simplification in simulation and the hydraulic resistance equation in analytical analysis. Additionally, micro-scale effects, such as interference between the fluorescent sample and the wall, wall friction, and surface tension, also contribute to errors in the micro-PIV measurements near the wall s [58, 59]. Therefore, this study employed the simulation to determine the velocity and hence the shear stress in each channel in the following sections.

### Shear influence on endothelial cells morphology

**Fig. 5:**
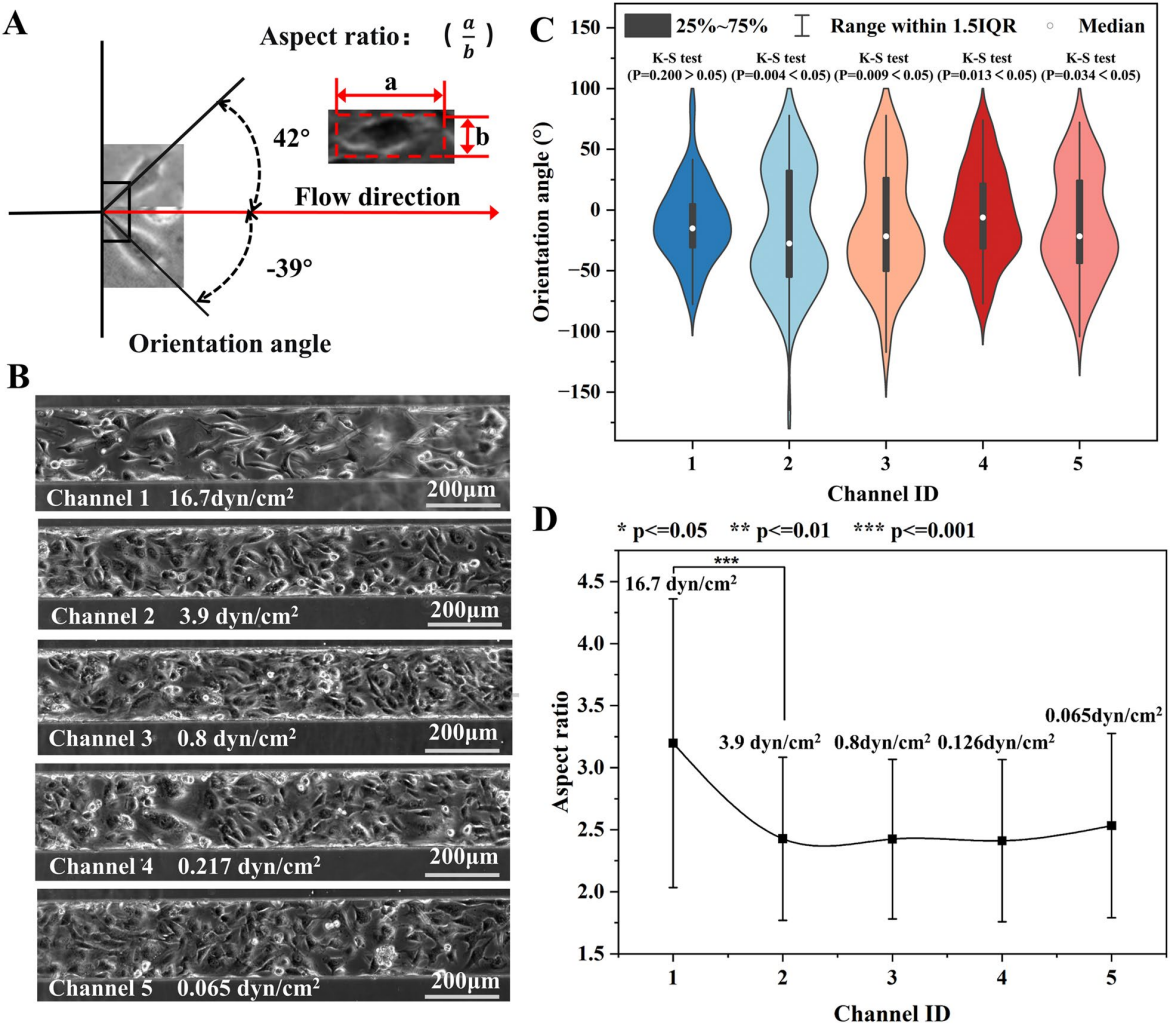
The morphology of HUVECs in devices without membrane deformation (Exponential decreasing shear profile). **A** The schematic diagram showing the orientation angle and aspect ratio of cells. **B** The phase contrast photos of HUVECs in 5 channels with various shear stress for 24h. **C** The orientation angle distribution of cells (N = 89–194). **D** The aspect ratio of cells in five branch channels with distinct shear stress

The devices generating both exponential (0.065–16.7 dyn/cm^2^) and linear (0.153–16.7 dyn/cm^2^) decreasing shear stress in 5 branch channels were employed to study the effect 24-h shear exposure on HUVECs. The shear stress in each channel and the relevant physiological condition were summarized in Additional file 1: Table S9. The effect of shear stress on cell morphology was quantitatively investigated by analyzing both the orientation angle and the aspect ratio of cells. As shown in Fig. 5A, the orientation angle referred to the accurate angle between the long axis of the cell and the flow direction. The aspect ratio represented the ratio between the length and the width of the cell.

Figure 5B illustrated the micro photos of HUVCEs in the shear stress of 0.065–16.7 dyn/cm^2^. The photos suggested that more cells realigned along the flow direction at higher shear stress. The orientation angle of cells (N = 89–187) in each channel was shown in Fig. 5C. The mean orientation angle of cells in Channel 1 (16.7 dyn/cm^2^) and Channel 2–5 was 26.4°and 34.6–42.0°, respectively. One-way ANOVA (P = 0.15–0.78) suggested no significantly difference between the mean orientation angles in the last four channels. Moreover, only the orientation angle of cells in Channel 1 was normally distributed according to K-S test (p = 0.2), while the others were randomly orientated (p = 0.004–0.034). Similarly, the mean aspect ratio of cells in Channel 1 and Channel 2–5 was 3.20 and 2.41–2.53, respectively. No significant difference was found in the aspect ratio in the last four channels (p = 0.10–0.96).

**Fig. 6:**
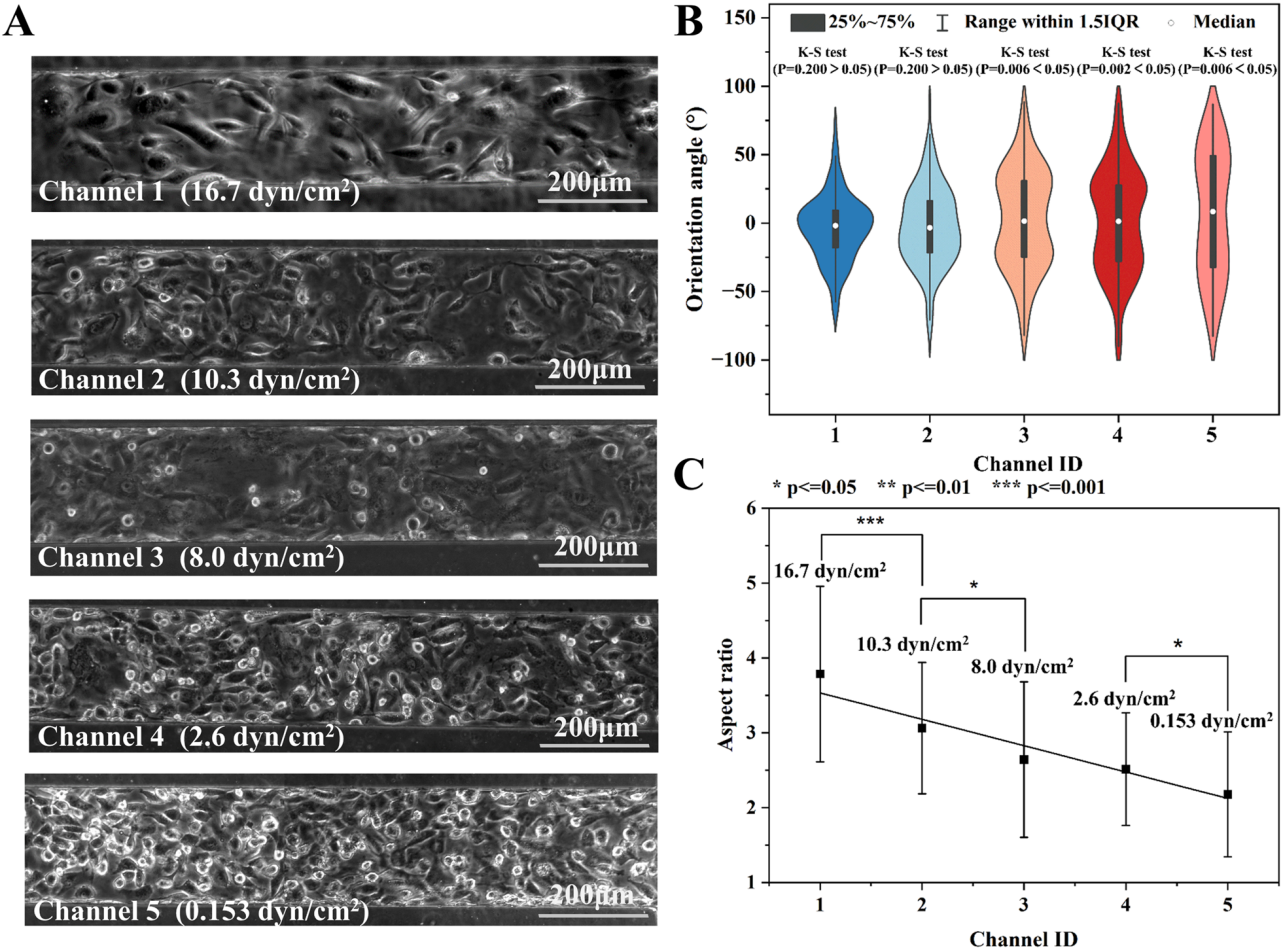
The morphology analysis of HUVECs exposed to shear stress for 24 h in devices with membrane deformation. **A** The micro photos of cell in five channels. **B** The orientation angle distribution and **C** the aspect ratio of cells (N = 122, 199, 187, 181, and 114) in five branch channels with distinct shear stress. The shear stress of 16.7 and 10.3 dyn/cm^2^ significantly affect cell orientation

Afterward, this paper showcased the precise regulation of shear stress in the five branch channels by controlling the membrane deformation, before studying the impact of large-range shear stress on cells. Figure 6A provided visual insights into the cellular response to the 24-h shear stress ranging from 0.153–16.7 dyn/cm^2^. Figure 6B illustrates the distribution of orientation angles for cells (N = 82–189) in each channel. The mean orientation angles for Channel 1–5 were 18.5°, 22.9°, 30.9°, 31.8°, and 40.4°, respectively. The K-S test revealed that the orientation angles of cells in Channel 1 and Channel 2 followed a normal distribution (p = 0.2 > 0.05). On the other hand, those in the remaining channels exhibited a random distribution (p < 0.05). Moreover, the average aspect ratios of cells in Channel 1–5 were 3.79, 3.06, 2.64, 2.51, and 2.18, respectively (Fig. 6C). Although there were large standard deviations, significant difference in aspect ratios was found for any two adjacent channels, except for those in channels 3 and 4.

Cell morphology is often studied using two primary parameters: cell aspect ratio and orientation angle, which provide insights into cell arrangement under shear stress. The latter one is particularly intuitive in characterizing cell organization. For example, the frequency of applied pulsating shear stress has a notable effect on the orientation angle of endothelial cells. Specifically, higher frequencies, such as 2 Hz, lead to a smaller positioning angle, causing the cells to align more towards the flow direction compared to lower frequencies of 0.25 and 0.75 Hz [60]. Moreover, the magnitude of the shear stress also plays a significant role in determining the distribution angle of endothelial cells. Applying shear stress ranging from 4 to 16 dyn/cm^2^ results in a gradual reduction of the orientation angle [61]. Interestingly, a larger shear stress of 10 dyn/cm^2^ tends to bring the positioning angle of the cells closer to a normal distribution in contrast to the smaller shear stress of 2 dyn/cm^2^ [33]. This phenomenon extends to other cell types as well, including fibroblasts [24, 29], stem cells [29], and osteoblasts [62]. Furthermore, the aspect ratio of a cell also responds to shear stress as a crucial cell size parameter. For instance, stem cells exhibit a certain level of shape response under a shear stress of 0.02 dyn/cm^2^, with a smaller cell aspect ratio observed at the shear stress of 0.022 dyn/cm^2^ [6]. Similarly, in the case of endothelial cells, the aspect ratio of the cells increases with higher shear stress ranging from 4 to 16 dyn/cm^2^ [61].

Therefore, it can be reasonably inferred that shear stress larger than 10 dyn/cm^2^ (equivalent to that in veins) had a significant impact on the cellular arrangement. Cells tended to align more closely with the direction of fluid shear stress, resulting in a normal distribution. Conversely, the low shear stress less than 8 dyn/cm^2^ (equivalent to that in the arteries) did not exert a significant influence on cell arrangement (as shown in Additional file 1: Figure S7). Moreover, the shear stress had a greater impact on the cell aspect ratio compared to its orientation angle. The obtained results exhibited similarity to the findings reported by DeStefano [61] and Arora [29] et al. In summary, the investigate on of cell morphology using cell aspect ratio and orientation angle, particularly under the influence of shear stress, provided valuable insights into the behavior of various cell types. These findings have implications for understanding cellular responses to mechanical stimuli and contribute to the broader understanding of cell biology.

### Blood vessel damage in thrombosis model

**Fig. 7:**
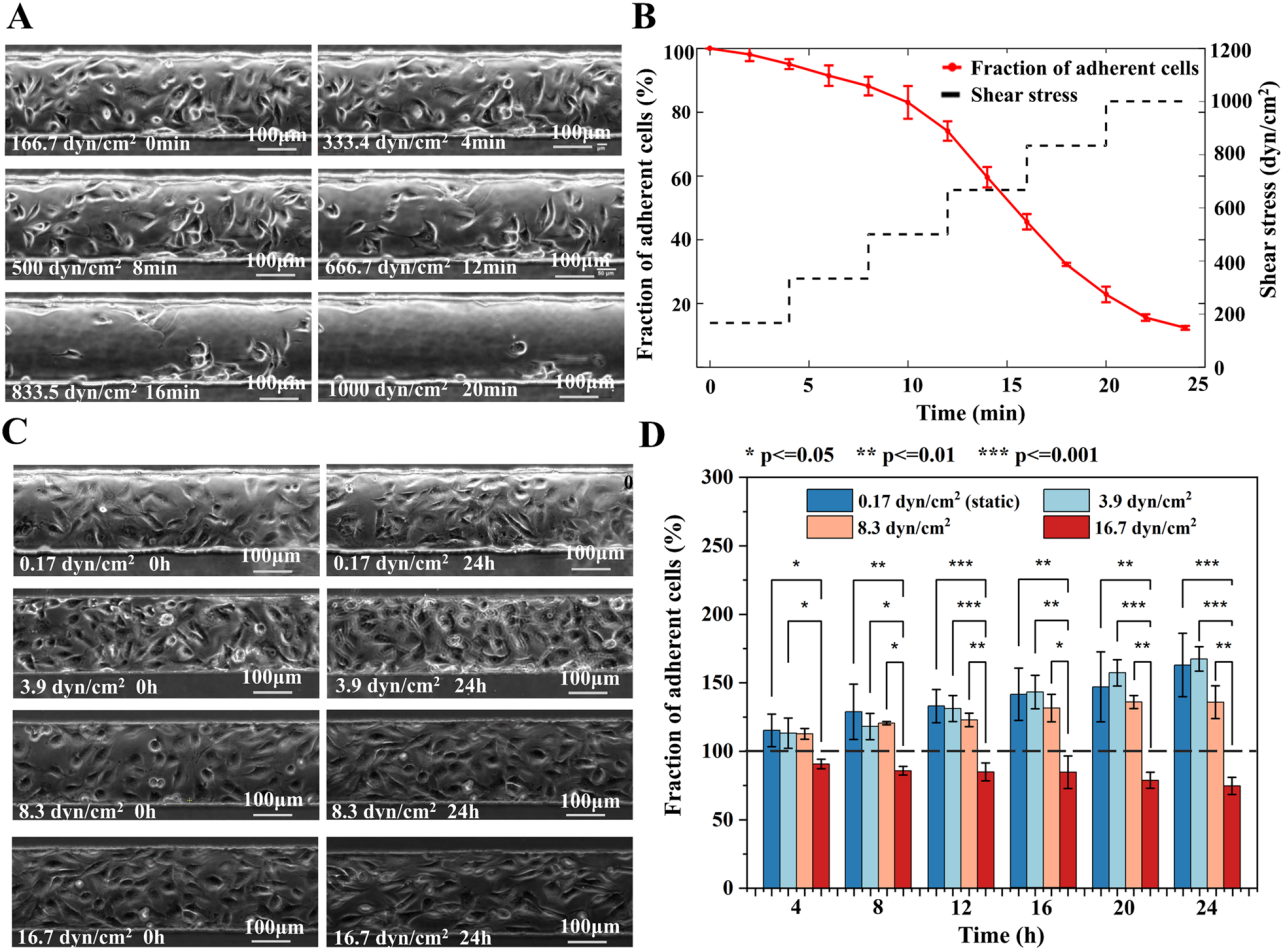
The cell attachment strength study. **A** The micro photos and **B** the percentage of HUVECs remaining adhered to the channel exposed to increasing shear stress for various time periods. Each shear stress lasted four minutes before further increase. **C** The micro photos and **D** number of adhered cells in channels with different shear stress (0.17, 3.9, 8.3, and 16.7 dyn/cm^2^) for upto 24 h

The adhesion of endothelial cells is critical for the physiological processes such as embryonic development, tissue homeostasis, and pathological conditions. Moreover, investigating the adhesion of cells under distinct magnitude and exposure time of shear stress has important implications for the long-term on-chip culturing of HUVECs. The short-term exposure of cells to high shear stress was firstly assessed to understand the possible influence of specific pathological flows (such as flow during thrombus formation) on endothelial cells. Figure 7A and Additional file 2: Video S1 showed the morphology change and detachment (decrease in number) of HUVECs as the shear stress raised from 166.7 to 1000 dyn/cm^2^. During the normal culture with media perfused at the shear stress of 0.17 dyn/cm^2^, the cells continuously divided and migrated on the channel floor to confluence. However, 5.0% cells especially in the middle of the channel were peeled off the floor and flushed away from the field of view when they were exposed to the shear stress of 166.7 dyn/cm^2^ for 4 min (Fig. 7B). While the shear stress was further increased to 333.4, 500, 666.7, 833.5, and 1000 dyn/cm^2^ and was maintained at each stage for 4 min, there were 88.2%, 74.0%, 45.7%, 22.9%, and 12.4% HUVECs remaining adherent on the floor of the channel, respectively. Research on arterial vascular disease indicates that abnormal constriction or narrowing of normal arteries and arterioles can significantly alter their hemodynamic conditions compared to healthy vessels. For instance, a 95% arterial constriction can generate shear stress exceeding 1000 dyn/cm^2^ [2]. The findings of this study demonstrate that under the influence of high shear stress, a considerable number of cells detach rapidly. Consequently, at sites where arteries undergo abnormal constriction, blood vessels may sustain certain levels of damage, increasing the risk of vessel rupture and bleeding [63, 64]. Furthermore, studies have revealed that elevated shear stress can stimulate platelet aggregation, further heightening the risk of stroke.

Additionally, the long-term exposure of cells to low shear stress was studied to understand the effect of human physiological flow on endothelial cells. Figure 7C showed the micro photos of cells cultured either before or 24 h after application of the shear stress from 0.17 to 16.7 dyn/cm^2^. As presented in Fig. 7D, Additional file 1: Figure S8 and Additional file 3: Video S2, when subjected to venous shear stresses of 0.17 dyn/cm^2^, 3.9 dyn/cm^2^ and 8.3 dyn/cm^2^ for 24 h, the cell quantity notably increased 62.7%, 67.2%, and 35.6%, respectively. However, the cell number in the field of view experienced a substantial decrease of 25.5% at the shear stress of 16.7 dyn/cm^2^. Additionally, the number of cells exposed the former two shear stress for the same time period has no significant difference (p = 0.45–0.90). This suggested that the shear increase did not affect the cell proliferation nor the adhesion. According to ANOVA, the fraction of adherent cells under venous shear stress of 0.17, 3.9, and 8.3 dyn/cm^2^ significantly differed from that under arterial shear stress of 16.7 dyn/cm^2^ within each time period (Fig. 7D). At 8.3 dyn/cm^2^ some of the cells were flushed away during division, but the spread cells still adhered to the floor and cell number still raised. Whereas, at the 16.7 dyn/cm^2^, most dividing cells and even some spread cells were detached by the flow which contributed to the obvious cell number reduction. Atherosclerosis commonly affects arterial branches. Under normal physiological conditions, the shear stress in arteries is approximately 10 dyn/cm^2^ [65]. The microfluidic chip proposed in this study simulates the flow conditions in the presence of a thrombus by modulating the film deformation, and the simulation results demonstrating the corresponding changes in shear stress are presented in Additional file 1: Figure S9. Additional file 1: Figure S9C illustrates that for patients with mild arterial blockage (around 20%), the shear stress reaches approximately 16.7 dyn/cm^2^. In clinical practice, mild stenosis may not exhibit obvious symptoms. However, our findings indicate that even under a prolonged shear stress of 16.7 dyn/cm^2^, the number of adherent cells decreases. Consequently, patients with mild blood vessel blockages are still at risk of blood vessel rupture and bleeding. Moreover, in the absence of clinical symptoms, the risk may be even greater.

Through the application of a microfluidic chip to generate shear stress, the researchers observed that both the magnitude of the shear stress and the surface density of fibronectin have an impact on cell adhesion. Specifically, when the shear stress reached 4000 dyn/cm^2^, approximately 80% of the cells detached from the surface within 4 min. Similarly, nearly 80% of the cells were detached from the glass within 24 min As the shear stress is gradually increased from 0 to 1600 dyn/cm^2^ [66]. Moreover, the mode of shear stress application impacts cell adhesion. At 10.7 dyne/cm^2^ within 24 h, a moderate and gradual increase (10% increase/h) resulted in 43% higher cell retention compared to a severe and rapid increase (15% increase/h) [67]. The researchers discovered that TNF-a plays a crucial role in cell adhesion under shear stress [68]. Additionally, enhanced fibronectin assembly leads to increased endothelial cells adhesion by upregulating integrin β1 and focal adhesion kinase expression. This highlights the underlying mechanisms of shear impact on cell adhesion [67].

Although the adhesion force of cells on the substrate may vary slightly due to the type of cells, substrate materials and the protein priming on the substrate. The detachment study is of great significance for evaluating the effect of shear stress on cell adhesion. These observations highlight the sensitivity of cell adhesion to the duration and magnitude of shear stress. Elevated shear stress and prolonged exposure time may result in substantial detachment of cells and lower growth rate. For example, the doubling time of cells at static culture was measured to be 19.71 ± 12.4 h [69], further supporting the notion that the two venous shear stress conditions did not significantly alter cell growth dynamics over the long term.

## Conclusions

This paper introduces a microfluidic shear stress generator with controllable multi-physics and tempo-spatial characteristics. The device is designed for studying in vitro physiological and thrombus models. Through precise control and stabilization of membrane deformation, we attain accurate manipulation of flow resistance in the microfluidic chip. The design of flow resistance within the chip is finalized based on fluid dynamics and hydraulic calculations. By altering membrane deflection in each channel and inlet flowrate, we predict thousands of shear stress combinations, thereby achieving the integration of pressure field and flow field and enabling spatiotemporal regulation of shear stress within the chip. Simultaneously, μPIV results indicate a robust consistency between the theoretical model predictions of linear and exponentially decreasing velocity and the actual flow field velocity. Following this, the generated shear stress was employed to simulate both physiological and pathological in vivo microenvironments. The impact of shear stress on cell adhesion and morphology was investigated. Our findings revealed a significant alteration in the aspect ratio and orientation angle of cells when subjected to a physiological shear stress exceeding 8.3 dyn/cm^2^ over a 24-h period. The orientation angle exhibited a normal distribution. In response to an instantaneous (4 min) application of extremely high pathological shear stress (ranging from 166.7 to 1000 dyn/cm^2^), there was a sharp decrease in the number of adherent cells, ranging from 12.4% to 88.2%. Moreover, continuous exposure to shear stress exceeding 8.3 dyn/cm^2^ for 24 h exhibited a notable impact on both cell growth and adhesion.

Shear stress has a direct influence on the morphology and arrangement of endothelial cells, thereby affecting vascular endothelial function and contributing to endothelial dysfunction [70]. Vascular endothelial dysfunction plays a crucial role in the early stages of atherosclerosis development [71]. The findings from this article reveal that shear stress above 8.3 dyn/cm^2^ significantly impact cell arrangement and aspect ratio, favoring alignment in the direction of flow. In contrast, lower shear stress result in a relatively disordered cell arrangement. This observation suggests that the atheroprotective effect observed under high shear stress may be attributed to the organized cell alignment in the direction of flow [72].

Diseases such as atherosclerosis-induced blood clots pose significant threats to both life and health. Research has indicated that in 95% of patients with severe blood vessel blockage caused by thrombus, the blockage can generate abnormal shear stress reaching up to 1000 dyn/cm^2^. Based on the experimental findings presented in this work, it is evident that cells detach from the substrate surface within a short period when exposed to such high shear stress (166.7 to 1000 dyn/cm^2^). Consequently, patients with severe thrombosis face a heightened risk of blood vessel damage at the thrombus site, which can lead to conditions like bleeding and stroke. Additionally, elevated shear stress can activate platelet aggregation, potentially exacerbating the blockage severity. Even for patients with mild blockage, our experimental results demonstrate that long-term exposure to micro-high shear stress (24h, 16.7 dyn/cm^2^) can also induce cell detachment. Considering that 50% of individuals with vascular occlusion exhibit no clinical symptoms, it is crucial to pay closer attention to the risks imposed by shear stress. The microfluidic chip described in this study offers a powerful tool for investigating the effects of shear stress on cells under physiological and pathological flow conditions. Its multi-physics functionality, spatial and temporal control, and dynamic generation of large-scale shear stress provide researchers with a reliable means to explore the impact of shear on cellular behavior. Importantly, this chip provides valuable support for understanding the underlying mechanisms involved in the formation of atherosclerosis, a significant cardiovascular disease. By simulating and studying shear-induced cellular responses, researchers can gain insights into the development and progression of atherosclerosis, ultimately contributing to advancements in disease understanding and potential therapeutic interventions.

The microfluidic chip in this article is constructed from PDMS, a material renowned for its exceptional biocompatibility. Consequently, following surface treatment, the chip can conducive for emulating the shear stress microenvironment experienced by adherent cells, including platelets, myoblasts, fibroblasts, stem cells, and diverse cancer cells. Simultaneously, it holds application potential in investigating the mechanical dynamics of suspended cells such as red blood cells and white blood cells. In conclusion, this microfluidic chip demonstrated exceptional compatibility, extending its utility beyond HUVECs to facilitate mechanical dynamics studies across various cell lines. The successful development of this device bears significant implications for advancements in cell biology, pathogenesis, and disease treatment.

### Supplementary Information

**Additional file 1.** Membrane deformation simulation. Flow resistance simulation. Theoretical study of the flow. Numerical study of the flow. Cell maintenance. **Figure S1.** The hydraulic computational design of microfluidic chips. **Figure S2.** Membrane deformation simulation. **Figure S3.** Flow resistance simulation. **Figure S4.** The velocity field at the entrance of the branch channel without the constriction. **Figure S5.** The velocity field at the exit of the branch channel without the constriction. **Figure S6.** The velocity field behind the constriction in the branch channel. **Figure S7.** The orientation angle distribution of cells under distinct shear stress. **Figure S8.** The growth curve adherent cells under various shear stress. **Figure S9.** The simulation results depict the flow field within the channel under various membrane deformation conditions, all subject to the same inlet flow conditions. **Table S1.** Mesh Independence Validation for membrane deformation simulation. **Table S2.** The relationship of membrane deformation and pressure. **Table S3.** Parameters in the mesh independence validation for flow resistance simulation. **Table S4.** The flow resistance of each part of the microfluidic chip. **Table S5.** The combination of membrane deformations and related shear stress range. **Table S6.** The same membrane deformation combination generates both maximum and minimum shear stress gradients. **Table S7.** The deformation of the membrane corresponding to different shear stress profiles. **Table S8.** The fitted model (y=ax^2^+bx+c) parameters for velocity in 5 channels (acquired by µPIV) without membrane deformation. **Table S9.** The Shear stress values generated by microfluidic chips in cell experiments.

**Additional file 2: Video S1.** The video showing the process of cell detachment under the shear stress increasing from 166.7 dyn/cm^2^ to 1000 dyn/cm^2^. Each shear stress was maintained for 4 minutes.

**Additional file 3: Video S2.** The video of the cells cultured either statically at 0.17 dyn/cm^2^ for 48 hours, or under the shear stress of 3.9 dyn/cm^2^, 8.3 dyn/cm^2^, and 16.7 dyn/cm^2^ for 24 hours each.

## Data Availability

The data that support the findings of this study are available from the corresponding author upon reasonable request.
