# Supplementary material for: A tempo-spatial controllable microfluidic shear-stress generator for in-vitro mimicking of the thrombus
Source: J Nanobiotechnology. 2024 Apr 17;22:187. doi: 10.1186/s12951-024-02334-6 (PMC11022418; doi:10.1186/s12951-024-02334-6)
Supplement: Supplementary file 1 — Additional file 1. Membrane deformation simulation. Flow resistance simulation. Theoretical study of the flow. Numerical study of the flow. Cell maintenance. Figure S1. The hydraulic computational design of microfluidic chips. Figure S2. Membrane deformation simulation. Figure S3. Flow resistance simulation. Figure S4. The velocity field at the entrance of the branch channel without the constriction. Figure S5. The velocity field at the exit of the branch channel without the constriction. Figure S6. The velocity field behind the constriction in the branch channel. Figure S7. The orientation angle distribution of cells under distinct shear stress. Figure S8. The growth curve adherent cells under various shear stress. Figure S9. The simulation results depict the flow field within the channel under various membrane deformation conditions, all subject to the same inlet flow conditions. Table S1. Mesh Independence Validation for membrane deformation simulation. Table S2. The relationship of membrane deformation and pressure. Table S3. Parameters in the mesh independence validation for flow resistance simulation. Table S4. The flow resistance of each part of the microfluidic chip. Table S5. The combination of membrane deformations and related shear stress range. Table S6. The same membrane deformation combination generates both maximum and minimum shear stress gradients. Table S7. The deformation of the membrane corresponding to different shear stress profiles. Table S8. The fitted model (y=ax2+bx+c) parameters for velocity in 5 channels (acquired by µPIV) without membrane deformation. Table S9. The Shear stress values generated by microfluidic chips in cell experiments. [file 12951_2024_2334_MOESM1_ESM.docx]

# Additional information

**A tempo-spatial controllable microfluidic shear-stress generator for in vitro study of the thrombus model**

Zhihang Yu^a^, Yiqun Chen^a^, Jingjing Li^b^, Chang Chen^a^, Huaxiu Lu^a^, Siyuan Chen^a^, Tingting Zhang^a^, Tianruo Guo^b^ Yonggang Zhu^a^, Jing Jin^a*^, Sheng Yan^c*^, and Huaying Chen^a^*

^a^ School of Mechanical Engineering and Automation, Harbin Institute of Technology, Shenzhen, Shenzhen 518055, China.

^b^ Graduate School of Biomedical Engineering, The University of New South Wales, NSW 2052, Sydney, Australia.

^c^ Institute for Advanced Study, Shenzhen University, Shenzhen 518060, China

Corresponding Author

*Email: [chenhuaying@hit.edu.cn](mailto:chenhuaying@hit.edu.cn); [shengyan@szu.edu.cn](mailto:shengyan@szu.edu.cn); jinjing2020@hit.edu.cn

Tel: +86 755 8615 3249.

## Content

**Membrane deformation simulation**……………………………..….……...….…..S4

**Flow resistance simulation**……………………………..…………….……...….…..S4

**Theoretical study of the flow**……………………………………..…………..……..S5

**Numerical study of the flow**………………………………….………….…....….…S6

**Cell maintenance**……………………..…………………………….…………….…S7

**Additional figures**………………………………..……………….…………....S8

Figure S1 The hydraulic computational design of microfluidic chips.……….....S8

Figure S2 Membrane deformation simulation……………………….……….....S9

Figure S3 Flow resistance simulation…………………………………..…...…S10

Figure S4 The velocity field at the entrance of the branch channel without the constriction……………………………………………………..……………...S11

Figure S5 The velocity field at the exit of the branch channel without the constriction.........................................................................................................S12

Figure S6 The velocity field behind the constriction in the branch channel…..S13

Figure S7 The orientation angle distribution of cells under distinct shear stress……………………………………………………………….…………..S14

Figure S8 The growth curve adherent cells under various shear stress………..S15

Figure S9 The simulation results depict the flow field within the channel under various membrane deformation conditions, all subject to the same inlet flow conditions……………………………………………………………..……….S16

**Additional Tables**……………………………..………………………..…..…S17

Table S1 Mesh Independence Validation for membrane deformation simulation……………………………………………………………………...S17

Table S2 The relationship of membrane deformation and pressure………..…. S18

Table S3 Parameters in the mesh independence validation for flow resistance simulation……………………………………………………………………...S19

Table S4 The flow resistance of each part of the microfluidic chip.…………..S20

Table S5 The combination of membrane deformations and related shear stress range………………………….………………………………………………..S21

Table S6 The same membrane deformation combination generates both maximum and minimum shear stress gradients…………………………………………...S22

Table S7 The deformation of the membrane corresponding to different shear stress profiles………………………………………….……………………….…….S23

Table S8 The fitted model (y=ax^2^+bx+c) parameters for velocity in 5 channels (acquired by µPIV) without membrane deformation ……………….……...….S24

Table S9 The Shear stress values generated by microfluidic chips in cell experiments……………………………………………………………………S25

**Reference**……………………………………...……………………...…...…...…...S27

## Membrane deformation simulation

In order to investigate membrane deformation, we focused on simulating a segment of the valve. The valve has a width of 200 μm and a length of 3 mm. We conducted separate simulation studies for PDMS materials with two different curing agent ratios: 10:1 and 25:1. The density of both materials is 970 kg/m³, and their Poisson's ratio is 0.49. The Young's modulus for the 10:1 ratio is 15 MPa, while for the 25:1 ratio, it is 10 MPa. For the boundary load of the membrane, we applied a uniform load. The load magnitudes were 69.90-226.63 mbar for the 10:1 ratio and 32.05-137 mbar for the 25:1 ratio. To conduct the simulation, we utilized a model and mesh configuration, as illustrated in Figure S2A. In order to determine the optimal mesh parameters, seven different mesh sizes were employed in this study (Table S1). For the material properties, a curing agent ratio of 10:1 PDMS was selected. A pressure of 226.63 mbar was applied to the membrane, and the resulting deformation of the membrane with different mesh is depicted in Figure S2B. Based on the results of the mesh independence verification, it was determined that mesh 5 provided the most suitable mesh resolution for the subsequent simulation. There are 32105 domain units, 3260 boundary elements, and 36 vertex unit. The average unit mass is 0.61.

Figures S2C and D depict the deformation of the X-Y interface and X-Z cross-section under varied pressure conditions for a PDMS film with a curing agent ratio of 10:1, while Figures S2F and G portray the corresponding deformation for a PDMS film with a curing agent ratio of 25:1. The figure reveals a parabolic deformation of the membrane on the X-Z section, aligning with the membrane deformation observed in the experiment illustrated in Figure 2C. On the X-Y cross-section, the membrane exhibits shape mutations at both ends, while the deformation in the middle part remains consistent. This observation is in line with the findings of Zhou, Y et al [1]. Upon comparing the experimental fitting curve of membrane deformation with pressure, it is evident that the simulated scatter points of membrane deformation, as shown in Figure S2E and H, closely align with the fitting curve. This serves as additional evidence affirming the accuracy of the experiment.

## Flow resistance simulation

For the case of valve flow resistance, a 3-dimensional model was used to simulate the flow in the valve section. The fluid material was water in the COMSOL material library. The temperature, density, and dynamic viscosity of the fluid were 20 °C (293.15 K), 1000 kg/m3, and 0.001 Pa∙s, respectively. No-slip boundary condition was selected for the walls of the microfluidic chip. The inlet velocity was 0.00278 m/s. The model and grid used in the simulation were shown in Figure S1A. To determine the optimal mesh parameters, seven different grid sizes were employed (as shown in Table S1). Mesh-independent verification was performed using a membrane deformed to 50 μm. Based on the results of the mesh independence verification, mesh with 2×10^6^ domain units provided the most suitable mesh resolution for the subsequent simulation (as shown in Figure S1B). The simulation results of valve section pressure under different valve deformation conditions were shown in Figure S1C. According to the relationship between pressure and flow resistance, the flow resistance under different deformation conditions were calculated and shown in Figure S1D.

## Theoretical study of the flow

Hydraulic analysis within a microfluidic chip parallels circuit analysis. Manipulating the flow resistance of branch channels enables precise flow control in the chip. Specifically, R_1_, R_3_, R_5_, R_7_, R_9_ and R_11_ represented the flow resistance of different regions on the main channel. They were proportional to the length according to Equation 1 if the cross section was given. Additionally, R_2_, R_4_, R_6_, R_8_, and R_10_ were the flow resistances of the five branch channels which were determined by simulation in the former section. The device design was illustrated in Figure S3A. The device comprises a single inlet, six outlets, and a total of 12 junctions. The equivalent circuit diagram for fluid hydraulic calculations was shown in Figure S2B. The flow resistance of each part on the microfluidic chip was been obtained by simulation (Table S4). The formulas for fluid hydraulic calculation using Matlab were:

$R_{t1}=R_{3}+\frac{R_{t2}R_{4}}{R_{t2}+R_{4}}$ (S1)

$R_{t2}=R_{5}+\frac{R_{t3}R_{6}}{R_{t3}+R_{6}}$ (S2)

$R_{t3}=R_{7}+\frac{R_{t4}R_{8}}{R_{t4}+R_{8}}$ (S3)

$R_{t4}=R_{9}+\frac{R_{10}R_{11}}{R_{10}+R_{11}}$ (S4)

$Q_{12}=Q_{23}+Q_{24}$ (S5)

$Q_{23}=\frac{Q_{24}R_{t1}}{R_{2}}$ (S6)

$Q_{24}=Q_{45}+Q_{46}$ (S7)

$Q_{45}=\frac{Q_{46}R_{t2}}{R_{4}}$ (S8)

$Q_{46}=Q_{67}+Q_{68}$ (S9)

$Q_{67}=\frac{Q_{68}R_{t3}}{R_{6}}$ (S10)

$Q_{68}=Q_{89}+Q_{810}$ (S11)

$Q_{89}=\frac{Q_{810}R_{t4}}{R_{8}}$ (S12)

$Q_{810}=Q_{1011}+Q_{1012}$ (S13)

$Q_{1011}=\frac{Q_{1012}R_{11}}{R_{10}}$ (S14)

By adjusting the channel length and considering the membrane deformation, it was possible to precisely control the flow and shear stress within the chip. Through mathematical calculations using MATLAB, the theoretical flow rate at each outlet was estimated.

## Numerical study of the flow

The Navier Stokes equation for laminar and steady-state flow was further simplified to:

$\boldsymbol{f}-\frac{1}{\rho}\nabla\boldsymbol{p}+\nu\nabla^{2}\boldsymbol{v=0}$ (S15)

For the case of no valve, a 2-dimensional model was used to simulate the flow in the chip. The fluid material is water in the COMSOL material library. The temperature, density, and dynamic viscosity of the fluid are 20°C (293.15K), 1000kg/m3, and 0.001Pa∙s, respectively. No-slip boundary condition was selected to the walls of the chip. The inlet velocity in the simulation was the same as the inlet velocity in the PIV experiment, which was 0.00278m/s (the flow rate is 100μL/h). The pressure of the six outlets was set to 0 Pa. The ultrafine mesh was selected. The largest and the smallest units were 1.39∙10^-5^ m and 1.61∙10^-7^ m, respectively. The simulation results were shown in Figures S4 and S5.

For the case of linear decrease profile simulation, a 3-dimensional model is used to simulate the flow in the chip. The deformation of the valves (50μm, 40μm, 0μm, 10μm, 50μm for Channel 1-5) of the five side channels was calculated according to the theoretical calculation. The physical parameters, flow parameters and boundary conditions of the fluid were the same as above. The simulation results are shown in Figure S6.

## Cell maintenance

The human umbilical vein endothelial cells (HUVECs) were employed. The composition of the media was are 89% Dulbecco's modified Eagle's medium (DMEM, Corning, USA), 10% fetal bovine serum (Every, Green, China), and 1% double antibody (Biosharp, Hefei, China). The HUVECs were cultured inside a Humidified incubator with 5% CO_2_ at 37±0.1℃. Once the culture was confluent, the cells were trypsinized and resuspended in the medium for following experiments.

## Additional figure

| 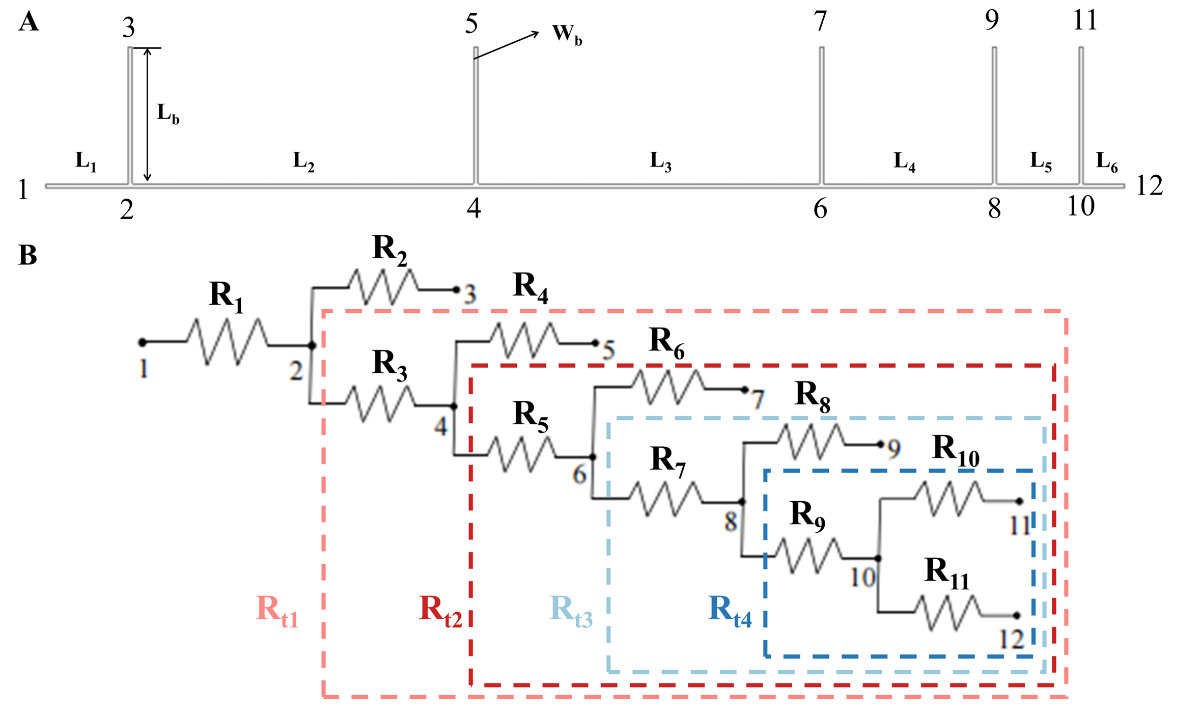 |
| --- |
| Figure S1 The hydraulic analysis of microfluidic chips. (A) Schematic diagram of the microchannel network; (B) The equivalent hydraulic circuit of the device for the hydraulic calculations. |

| 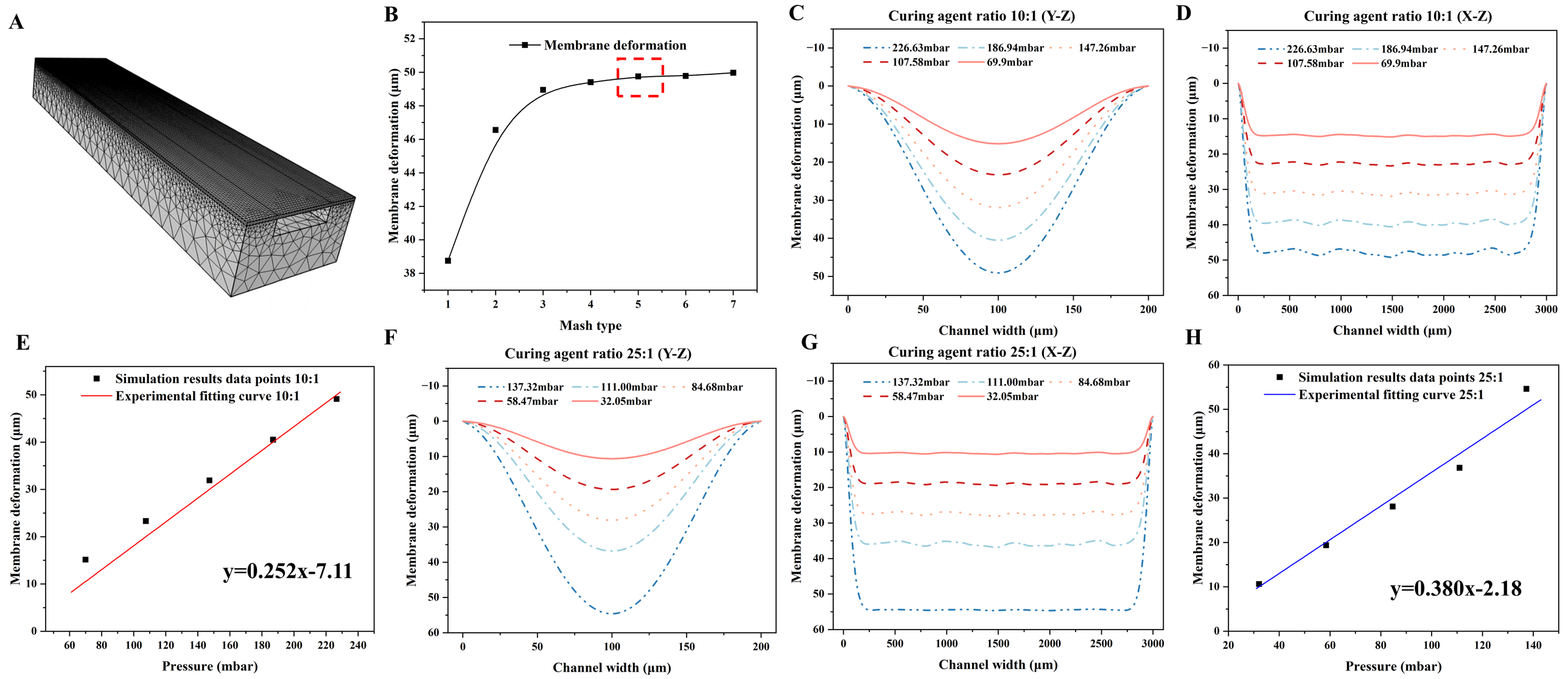 |
| --- |
| Figure S2 Membrane deformation simulation. (A) Valve deformation simulation model and mesh; (B) The mesh independence verification results for the simulation of membrane deformation (curing agent ratio 10:1, membrane force 226.63 mbar); The deformation of membrane with curing agent ratio 10:1 (C)＆(D) and 25:1 (F)＆(G); (E) and (H) depict the comparison between simulation results and experimental fitting curves illustrating the relationship between membrane deformation and pressure for PDMS membrane with curing agent ratios of 10:1 and 25:1. |

| 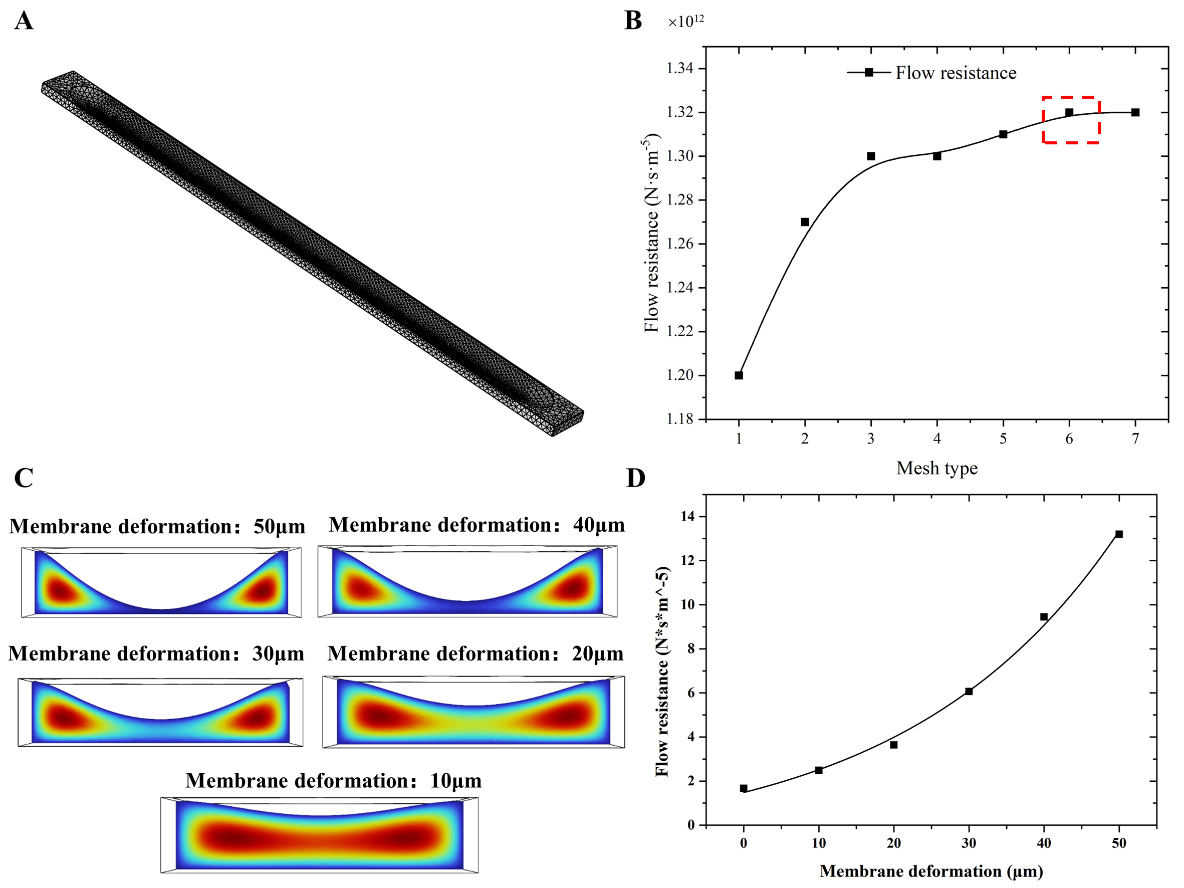 |
| --- |
| Figure S3 Flow resistance simulation. (A) Flow resistance simulation model and mesh; (B) The mesh-independent verification for flow resistance; (C) The flow velocity field through the cross section of the constriction; (D) The flow resistance at various membrane deformation. |

| 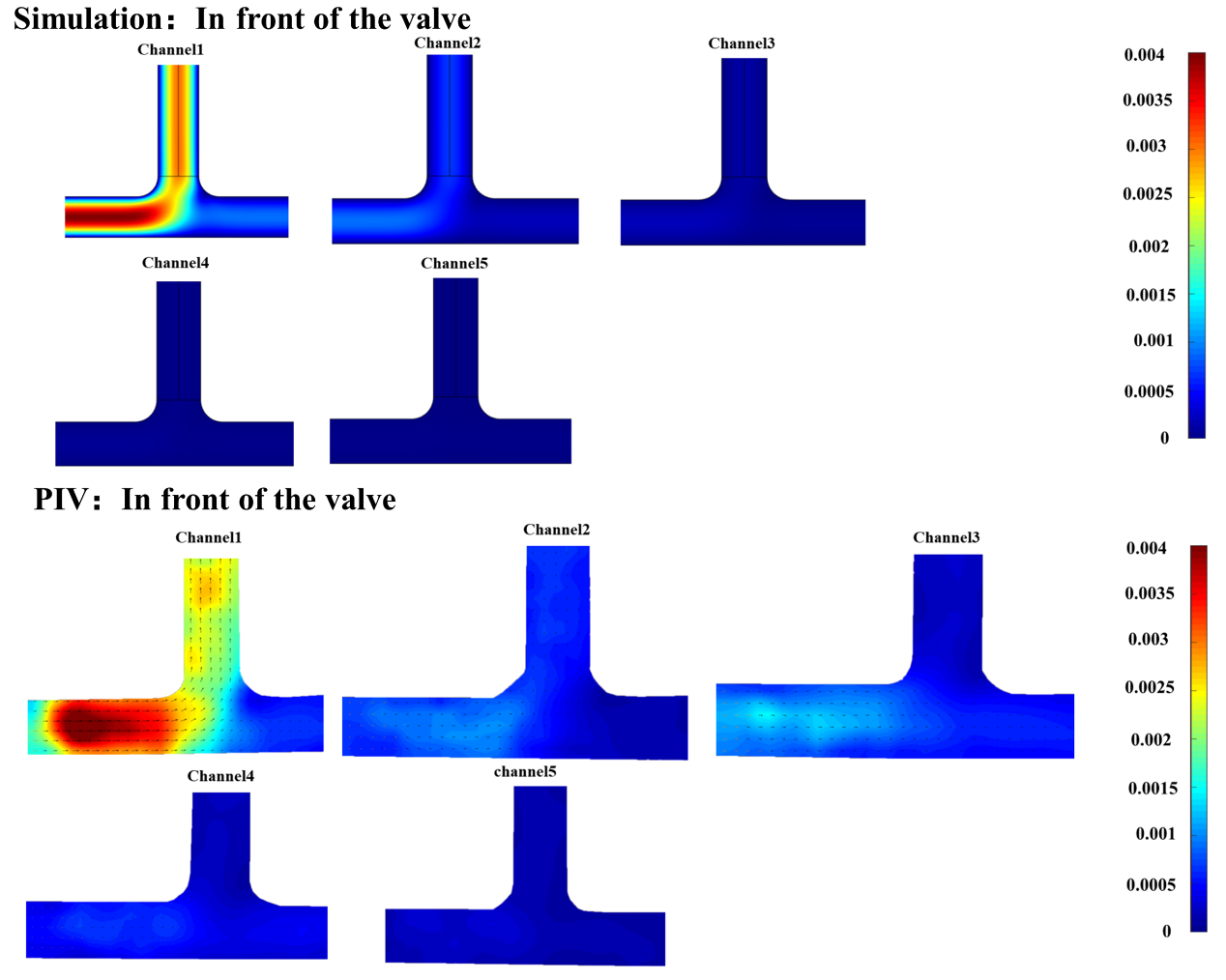 |
| --- |
| Figure S4. The velocity field (acquired by simulation and µPIV measurement) at the entrance of the branch channels without constriction. |

| 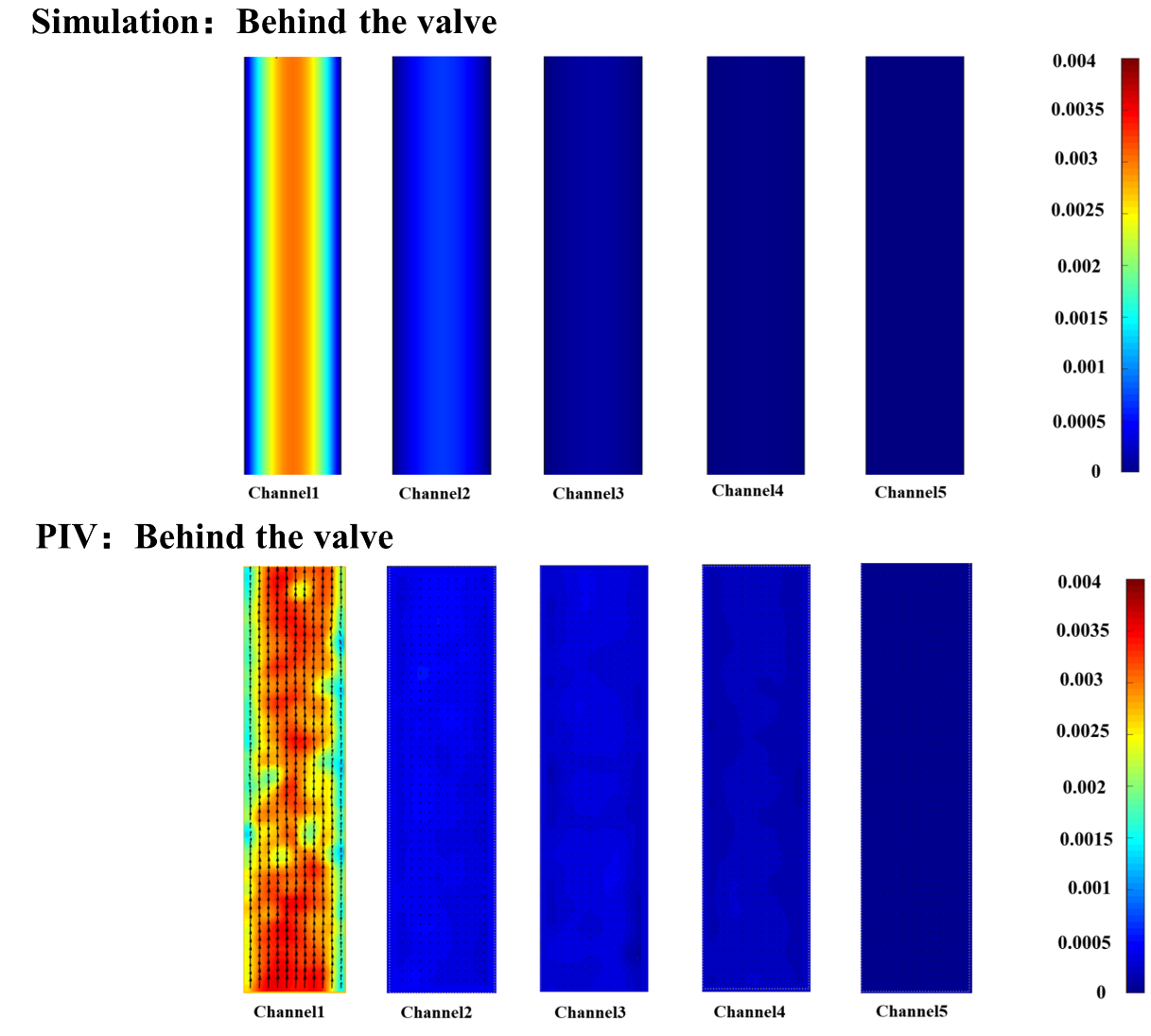 |
| --- |
| Figure S5. The velocity field (acquired by simulation and µPIV measurement) at the exit of the branch channel without valve. |

| 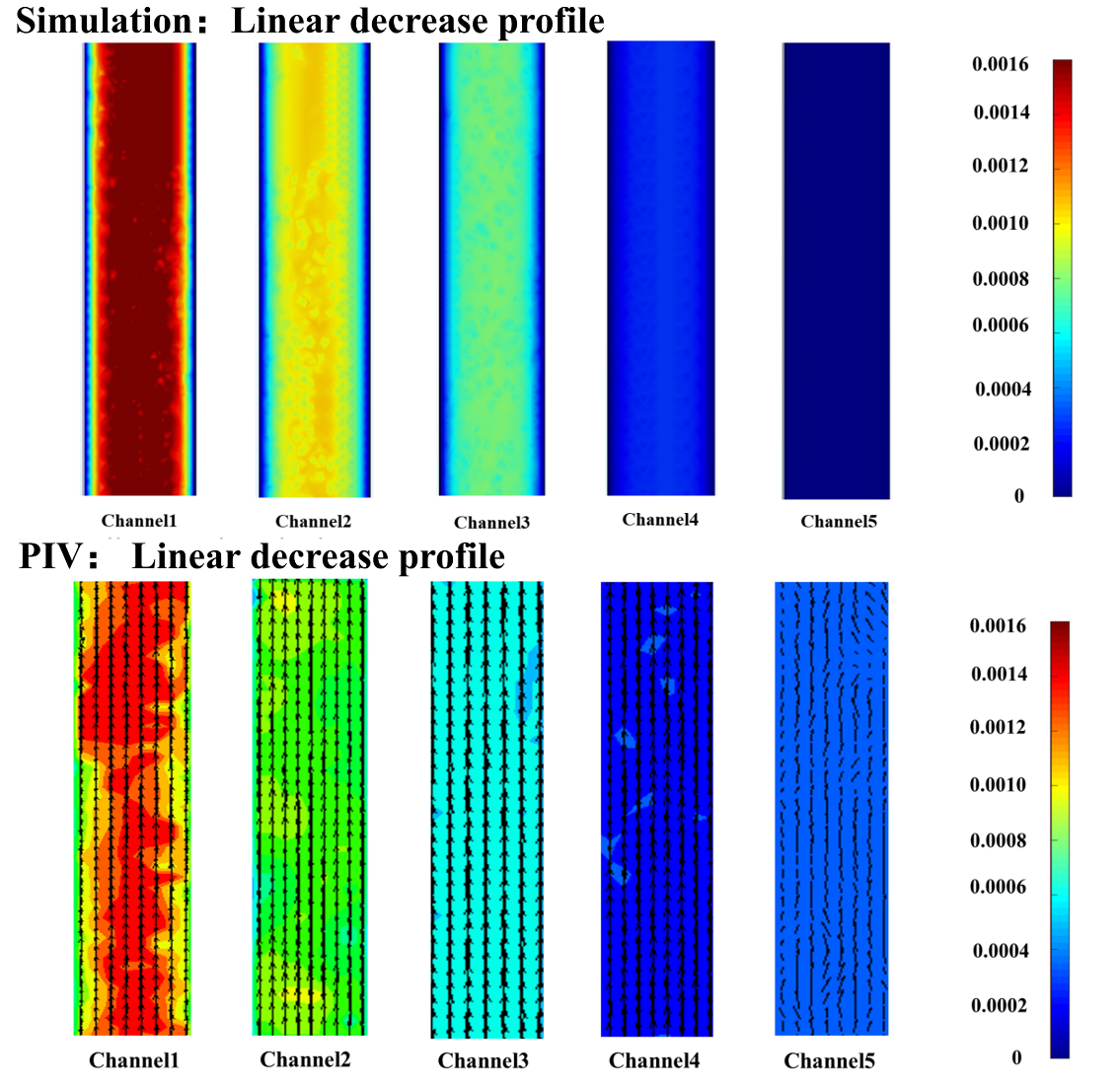 |
| --- |
| Figure S6. The velocity field (acquired by simulation and µPIV measurement) behind the valve in the branch channel. |


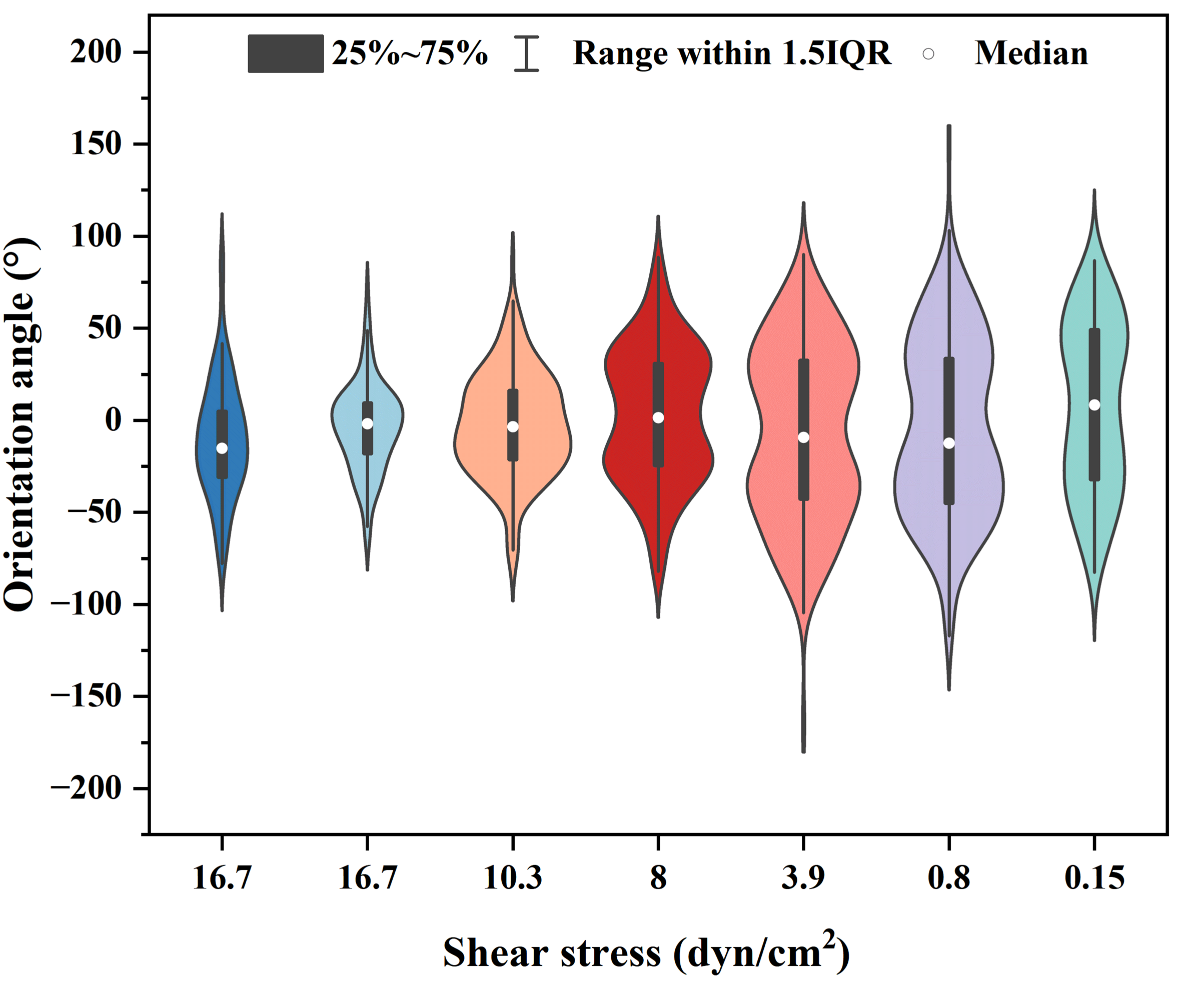


Figure S7 The orientation angle distribution of cells under distinct shear stress.


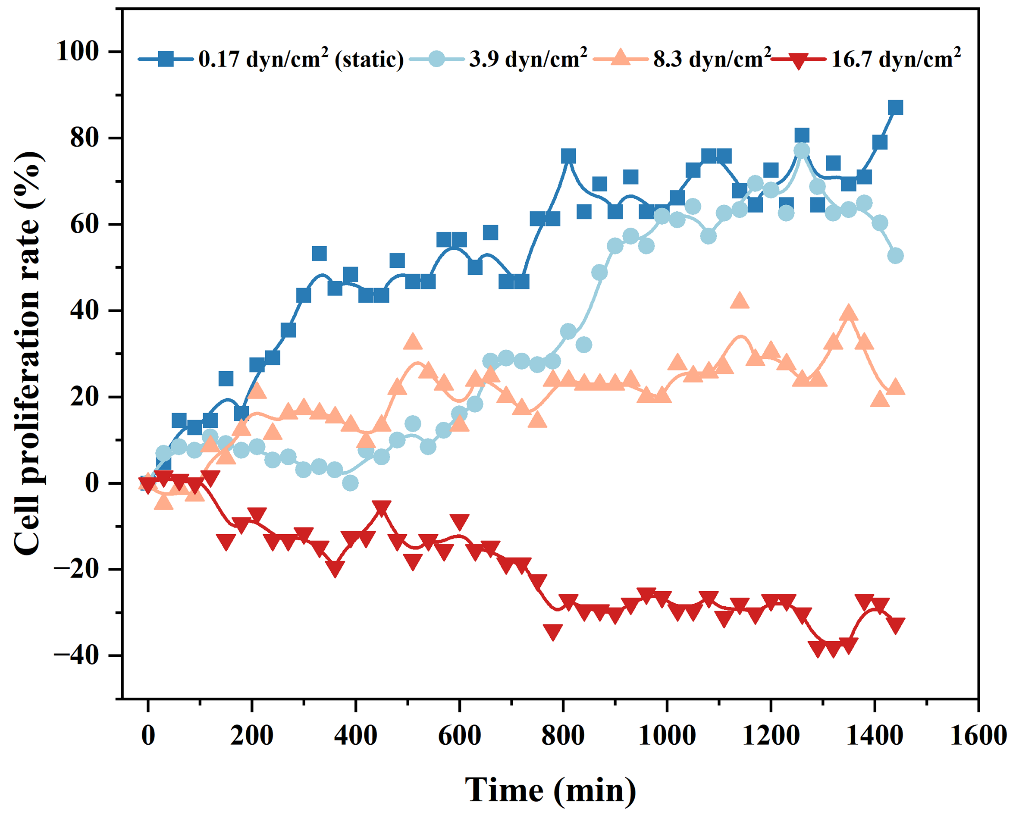


Figure S8 The growth curve adherent cells under various shear stress.

| 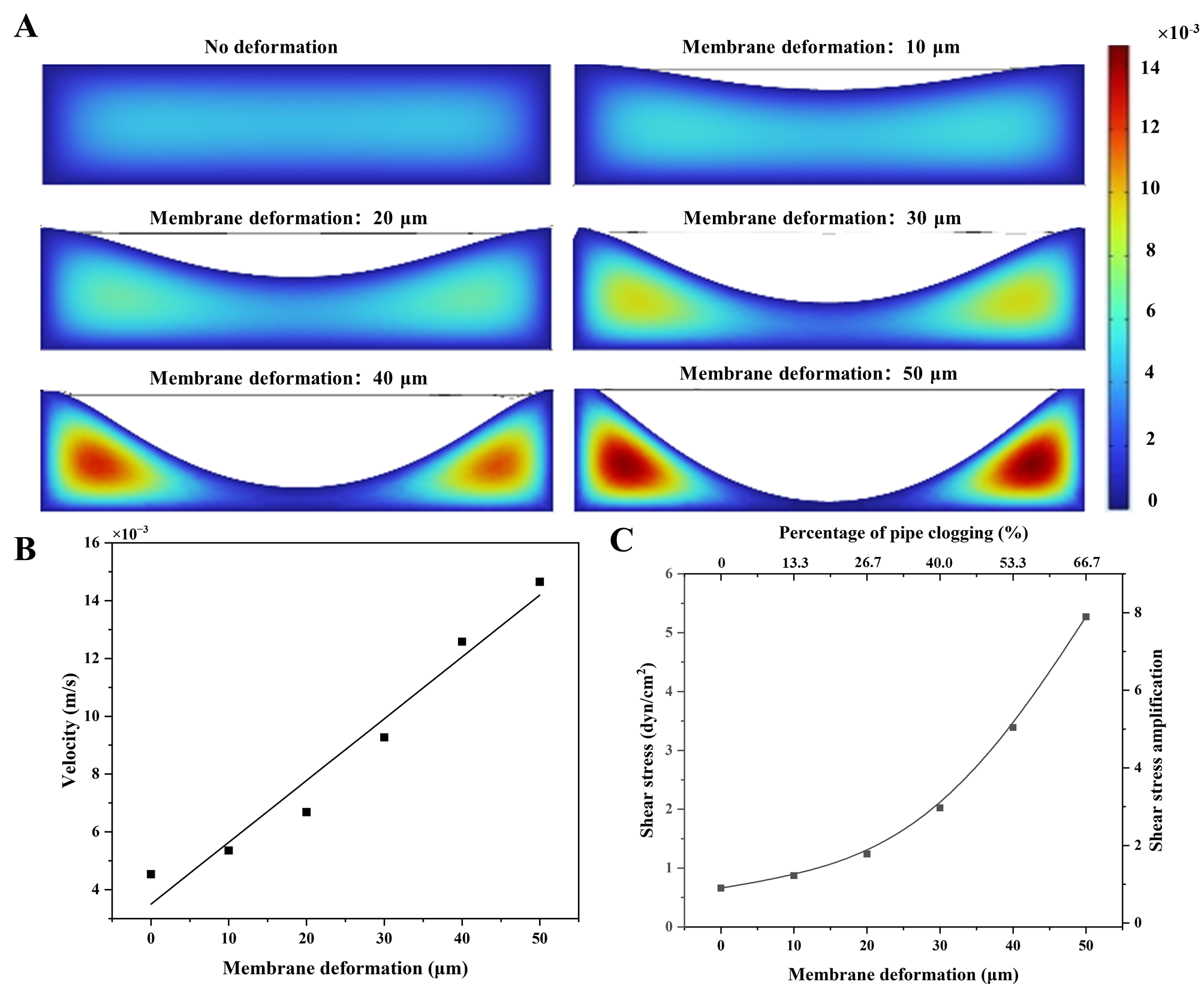 |
| --- |
| Figure S9 The simulation results depict the flow field within the channel under various membrane deformation conditions, all subject to the same inlet flow conditions. |

## Additional Table

Table S1 Mesh Independence Validation for membrane deformation simulation

| Mesh type | Maximum mesh size (mm) | Minimum mesh size (mm) | Average mesh quality |
| --- | --- | --- | --- |
| 1 | 0.608 | 0.1280 | 0.35 |
| 2 | 0.480 | 0.0896 | 0.38 |
| 3 | 0.320 | 0.0576 | 0.46 |
| 4 | 0.256 | 0.0320 | 0.51 |
| 5 | 0.176 | 0.0128 | 0.61 |
| 6 | 0.112 | 0.0048 | 0.67 |
| 7 | 0.064 | 0.0006 | 0.67 |

Table S2 The relationship between the membrane deformation and activation pressure

| Membrane deformation(μm) | Pressure(mbar)  (Curing agent ratio at 10:1) | Pressure(mbar)  (Curing agent ratio at 25:1) |
| --- | --- | --- |
| 10 | 69.90 | 32.05 |
| 20 | 107.58 | 58.37 |
| 30 | 147.26 | 84.68 |
| 40 | 186.94 | 111.00 |
| 50 | 226.63 | 137.32 |

Table S3 Parameters in the mesh independence validation for flow resistance simulation

| Mesh type | Maximum mesh size (mm) | Minimum mesh size (mm) | Average mesh quality |
| --- | --- | --- | --- |
| 1 | 0.0282 | 0.00868 | 0.63 |
| 2 | 0.0217 | 0.00651 | 0.65 |
| 3 | 0.0145 | 0.00434 | 0.67 |
| 4 | 0.0115 | 0.00217 | 0.67 |
| 5 | 0.0080 | 0.00087 | 0.66 |
| 6 | 0.0050 | 0.00033 | 0.66 |
| 7 | 0.0028 | 0.00004 | 0.66 |

Table S4 The flow resistance of each part of the microfluidic chip (×10^12^ N·s·m^-5^)

| Membrane deformation (μm) | Branch channel flow resistance (valve only) | | Branch channel flow resistance | | Main channel flow resistance |
| --- | --- | --- | --- | --- | --- |
| 0 | | 1.67 | | 4.46 | R_1_=2.78 |
| 10 | | 2.49 | | 5.27 | R_3_=11.14 |
| 20 | | 3.64 | | 6.42 | R_5_=11.14 |
| 30 | | 6.06 | | 8.84 | R_7_=5.57 |
| 40 | | 9.45 | | 12.24 | R_9_=2.78 |
| 50 | | 13.20 | | 16.00 | R_11_=1.39 |

Table S5 The combination of membrane deformations and related shear stress range

| Condition | Parameters | Channel1 | Channel2 | Channel3 | Channel4 | Channel5 |
| --- | --- | --- | --- | --- | --- | --- |
| Maximum shear stress (inlet flow: 737.0 μL/min) | Deformation (μm) | 0 | 50 | 50 | 50 | 50 |
|  | Flow rate (μL/min) | 598.8 | 70.8 | 37.0 | 13.6 | 4.3 |
|  | Shear stress (dyn/cm^2^) | **1000** | 118.2 | 61.7 | 22.7 | 7.1 |
| Minimum shear stress (inlet flow: 737.0 μL/min) | Deformation (μm) | 0 | 0 | 0 | 0 | 50 |
|  | Flow rate (μL/min) | 563.7 | 131.4 | 26.6 | 7.3 | 0.6 |
|  | Shear stress (dyn/cm^2^) | 941.3 | 219.4 | 44.4 | 12.3 | **1.08** |

Table S6 The same membrane deformation combination generates both maximum and minimum shear stress gradients

| Condition | Parameters | Channel1 | Channel2 | Channel3 | Channel4 | Channel5 |
| --- | --- | --- | --- | --- | --- | --- |
| Maximum shear stress (inlet flow: 783.0 μL/min) | Deformation (μm) | 0 | 0 | 0 | 0 | 50 |
|  | Flow rate (μL/min) | 598.8 | 139.6 | 28.3 | 7.8 | 0.7 |
|  | Shear stress (dyn/cm^2^) | 1000 | 233. 1 | 47.2 | 13.0 | 1.1 |
| Minimum shear stress (inlet flow: 1095 μL/min) | Deformation (μm) | 50 | 50 | 50 | 50 | 0 |
|  | Flow rate (μL/min) | 598.8 | 253.9 | 85.3 | 30.5 | 30.2 |
|  | Shear stress (dyn/cm^2^) | 1000 | 424.0 | 142.4 | 50.9 | 50.4 |

Table S7 The deformation of the membrane corresponding to different shear stress profiles

| Line type | Channel Number | Channel | Channel2 | Channel3 | Channel4 | Channel5 |
| --- | --- | --- | --- | --- | --- | --- |
| Square wave | Membrane deformation (μm) | 50 | 0 | 50 | 50 | 50 |
|  | Flow rate (μL/h) | 47.77 | 41.28 | 2.52 | 0.93 | 0.29 |
| Linear | Membrane deformation (μm) | 50 | 40 | 10 | 0 | 50 |
|  | Flow rate (μL/h) | 52.51 | 25.54 | 11.40 | 3.73 | 0.33 |
| Sine | Membrane deformation (μm) | 50 | 0 | 0 | 50 | 10 |
|  | Flow rate (μL/h) | 77.90 | 10.34 | 12.13 | 1.24 | 0.39 |
| Jaggies | Membrane deformation (μm) | 0 | 50 | 0 | 50 | 50 |
|  | Flow rate (μL/h) | 80.67 | 9.05 | 11.08 | 1.13 | 0.36 |

Table S8 The fitted model (y=ax^2^+bx+c) parameters for velocity in 5 channels (acquired by µPIV) without membrane deformation

| Channel ID | a | b | c | R^2^ |
| --- | --- | --- | --- | --- |
| 1 | -339.4 | 67.9 | 0 | 0.94 |
| 2 | -48.6 | 9.7 | 0 | 0.79 |
| 3 | -38.3 | 7.7 | 0 | 0.87 |
| 4 | -23.9 | 4.8 | 0 | 0.88 |
| 5 | -2.9 | 0.59 | 0 | 0.93 |

Table S9 The Shear stress values generated by microfluidic chips in cell experiments

| Shear stress pfrofile | Channel Number | Channel | | Channel2 | | Channel3 | Channel4 | Channel5 |
| --- | --- | --- | --- | --- | --- | --- | --- | --- |
| Linear decreasing profile | Membrane deformation (μm) | 50 | 40 | | 10 | | 0 | 50 |
|  | Shear stress (dyn/cm^2^) | 16.7 | 10.3 | | 8.0 | | 2.6 | 0.153 |
| Exponential decreasing profile | Membrane deformation (μm) | 0 | 0 | | 0 | | 0 | 0 |
|  | Shear stress (dyn/cm^2^) | 16.7 | 3.9 | | 0.8 | | 0.126 | 0.065 |

## Reference

1. Zhou Y, Yu ZB, Wu M, Lan YW, Jia CP, Zhao JL: **Single-cell sorting using integrated pneumatic valve droplet microfluidic chip.** *Talanta* 2023, **253**.
